# Degradation Mechanisms Associated with Electron‐Blocking Layers in Inverted Perovskite Solar Cells

**DOI:** 10.1002/advs.75170

**Published:** 2026-04-07

**Authors:** Xiongzhuo Jiang, Jie Zeng, Kun Sun, Simon Alexander Wegener, Zerui Li, Guangjiu Pan, Sarathlal Koyiloth Vayalil, Matthias Schwartzkopf, Baomin Xu, Peter Müller‐Buschbaum

**Affiliations:** ^1^ Department of Materials Science and Engineering Southern University of Science and Technology Shenzhen China; ^2^ TUM School of Natural Sciences Department of Physics Chair for Functional Materials Technical University of Munich Garching Germany; ^3^ Shenzhen BTR New Energy Technology Institute Co., Ltd Shenzhen Guangdong China; ^4^ Department Perovskite Tandem Solar Cells Helmholtz‐Zentrum Berlin für Materialien und Energie GmbH Berlin Germany; ^5^ Deutsches Elektronen‐Synchrotron DESY Hamburg Germany; ^6^ Applied Sciences Cluster University of Petroleum and Energy Studies UPES Dehradun Uttarakhand India

**Keywords:** degradation mechanism, electron‐blocking layer, inverted perovskite solar cells, operando measurement, strain evolution

## Abstract

The operational stability, especially under thermal cycling conditions, of perovskite solar cells (PSCs) based on different electron‐blocking layers (EBLs) is still missing. Here, we investigate the device performance of PSCs based on representative EBLs of NiO_x_, PTAA, and 2PACz as well as the degradation mechanism using *operando* grazing incidence wide‐angle X‐ray (GIWAXS) measurements. 2PACz‐based devices achieve an impressive PCE of 25.74% (certified 25.34%), which is significantly higher than 23.35% for NiO_x_‐based and 24.57% for PTAA‐based PSCs, attributed to the reduced trap density, prolonged charge carrier lifetime, and suppressed non‐radiative recombination. In addition, the 2PACz‐based devices exhibit excellent stability under light soaking (>1500 h of *T_90_* lifetime) and rapid solar‐thermal cycling conditions (no PCE degradation). *Operando* GIWAXS demonstrates that this stability originates from a relatively lower lattice distortion at both the buried side and the surface side of the perovskite layer of the 2PACz‐based device under the temperature variation during solar‐thermal cycling conditions. This work provides experimental evidence for the rational selection of EBL materials and highlights the interface regulation for constructing highly stable perovskite devices.

## Graphical abstract

The effects of the three typical EBLs NiO_x_, PTAA, and 2PACz on the photovoltaic performance and degradation mechanisms are investigated under light soaking and solar‐thermal cycling conditions of corresponding PSCs, demonstrating that 2PACz is the best EBL due to the better interface contact and lower lattice distortion.

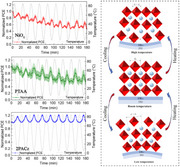

## 1 Introduction

Perovskite materials have received tremendous attention in photovoltaic devices as light absorbers due to their excellent properties, including a tunable bandgap [1, 2], a high absorption coefficient [3], a low non‐radiative recombination [4], a long charge carrier lifetime [5], and a high mobility of charge carriers [6]. Perovskite solar cells (PSCs), especially inverted PSCs, have realized significant progress in certified power conversion efficiency (PCE) recently, reaching 27% [7], which can be attributed to the improvement in charge carrier blocking materials selection [8, 9, 10], the perovskite crystallization control [11, 12], passivation strategy [13, 14, 15], and interface modification [16, 17]. For inverted PSCs, the electron‐blocking layer (EBL) between the substrate and the perovskite layer is responsible for hole extraction and transport, which directly impacts the energy level alignment, charge carrier dynamics, and interface stability [18]. However, the stability issue continues to hinder the commercialization of PSCs. In this regard, the ideal EBL should not only have a high hole mobility and a suitable energy level but also show few interface defects and high stability [19].

Nowadays, NiO_x_, PTAA, and 2PACz are regarded as promising EBL materials for inverted PSCs, representing inorganic, polymer, and self‐assembled monolayer (SAM) materials, respectively. NiO_x_ has received significant research interest as an inorganic material due to its excellent stability and low cost [20, 21]. However, the high interface defect density limits its further application [22]. The polymer PTAA can deliver impressive efficiency benefits from its high hole mobility and suitable energy level, but its insufficient wettability and high cost limit large‐scale fabrication [23, 24, 25]. 2PACz, as a typical SAM material in inverted PSCs, has attracted tremendous attention in recent years, due to its properties in hole extraction and defect passivation [26, 27]. However, there are rare reports systematically investigating the effect of these three typical EBL materials on the photovoltaic performance and stability in the same device architecture, especially the degradation mechanism induced by these EBLs under thermal cycling (ISOS‐T) or solar‐thermal cycling (ISOS‐LT), which is proposed in the International Summit on Organic Photovoltaic Stability (ISOS) protocols [28].

To gain a deep understanding of the effects of NiOx, PTAA, and 2PACz on PSCs, this work systematically investigates device performance based on these three different EBLs and their degradation mechanisms under thermal cycling using advanced *operando* grazing incidence wide‐angle X‐ray scattering (GIWAXS) measurements. As a result, the 2PACz‐based device achieves an impressive PCE of 25.74% (certified 25.34%), which is significantly higher than 23.35% for NiO_x_‐based and 24.57% for PTAA‐based devices, benefiting from the significant improvement in open circuit voltage (*V_OC_*) and a slight increase in FF, attributed to the reduced trap density, prolonged charge carrier lifetime, and suppressed non‐radiative recombination. The 2PACz‐based device exhibits excellent stability under light soaking (>1500 h of *T_90_* lifetime) and rapid solar‐thermal cycling conditions (no PCE degradation). The *operando* GIWAXS measurement reveals that even though the reversible phase transition and reduced crystallinity are the common reasons for the degradation of PSCs [29, 30], they are not the main reason for the stability difference among NiO_x_‐, PTAA‐, and 2PACz‐based devices, due to almost the same perovskite structure evolution among them during the thermal cycling measurement. However, the lattice distortion induced by temperature variation results in a stability difference among NiO_x_‐, PTAA‐, and 2PACz‐based devices. Compared to NiO_x_‐ and PTAA‐based devices, the 2PACz‐based device exhibits a relatively lower lattice distortion at both the buried side and the surface side under temperature variations during solar‐thermal cycling conditions.

## 2 Results and Discussion

### 2.1 Photovoltaic Performance of PSCs

**FIGURE 1:**
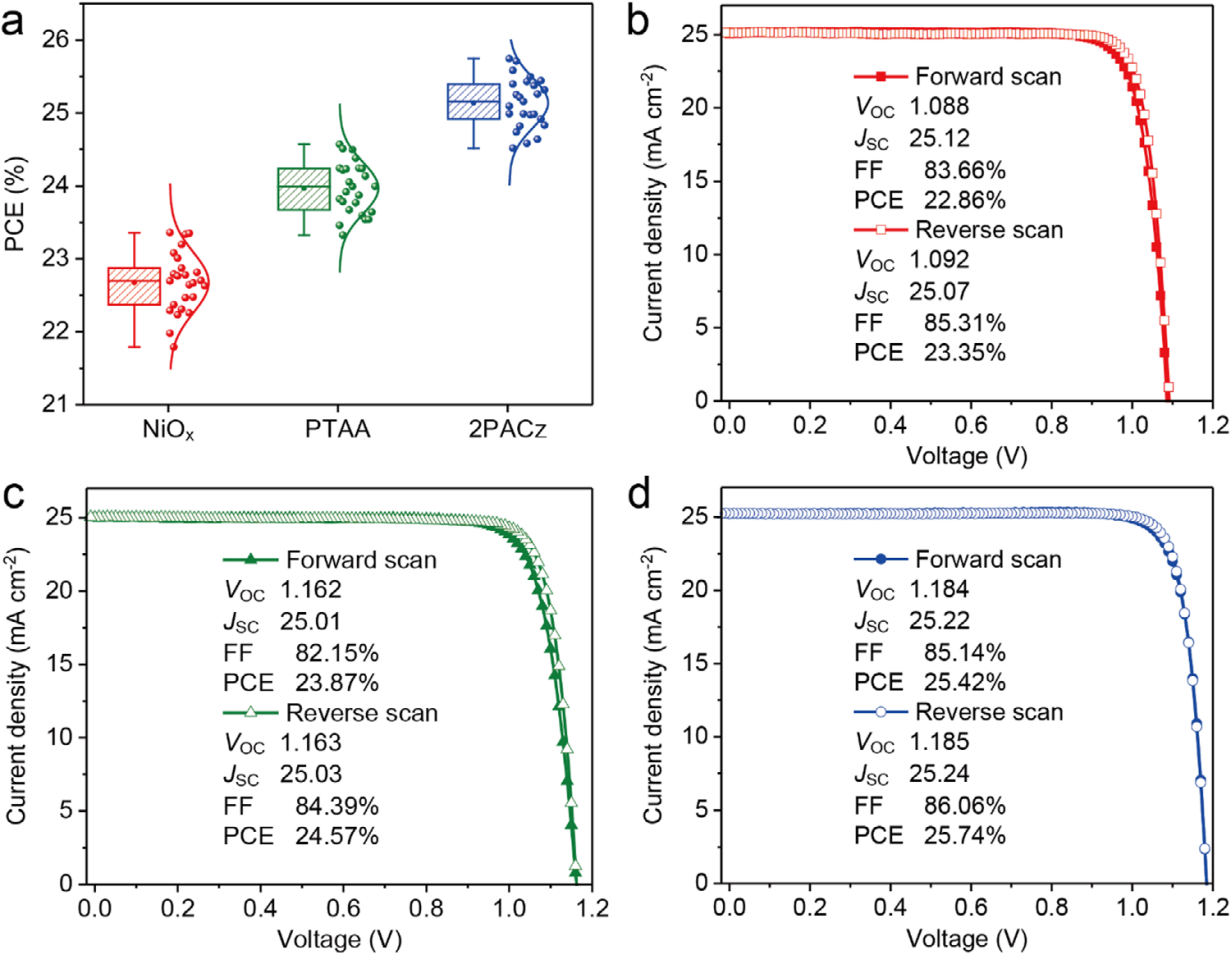
Photovoltaic performance of PSCs. (a) Box plot of the PCE distribution for PSCs based on NiO_x_, PTAA, and 2PACz. The champion *J‐V* curves of PSCs based on (b) NiO_x_, (c) PTAA, and (d) 2PACz with both reverse and forward scan directions within the voltage region −0.2 to 1.2 V.

To systematically investigate the effect of the different EBLs NiO_x_, PTAA, and 2PACz on the photovoltaic performance and stability of inverted PSCs, the same device architecture of ITO/EBLs/Perovskite/Passivation/C_60_/BCP/Ag (Figure S1) is used for all EBL materials. The composition of the investigated perovskite is Cs_0.05_FA_0.85_MA_0.1_PbI_3_. The PCE distributions for the device based on NiO_x_, PTAA, and 2PACz are shown in Figure 1a, which was collected from 26 individual devices for each EBL. Obviously, the 2PACz‐based device exhibits the highest average PCE of 25.14% ± 0.34%. In contrast, the device based on NiO_x_ shows the lowest average PCE of 22.68% ± 0.41% with a slightly broader distribution. The PTAA‐based device achieves an average PCE of 23.97% ± 0.35%. The corresponding distributions of *V_OC_*, *J_SC_*, and fill factor (FF) values are shown in Figure S2, which indicates that the main difference in PCE values among these three device types is attributed to the differences in *V_OC_*. The corresponding *J‐V* curves of the champion devices for each EBL further verify this result (Figure 1b–d). The PCE of the champion device based on 2PACz reaches 25.74% under reverse scan, with a *V_OC_* of 1.185 V, *J_SC_* of 25.24 mA cm^2^, and a FF of 86.06%, which is significantly higher than the NiO_x_‐based device showing a PCE of 23.35%, a *V_OC_* of 1.092 V, *J_SC_* of 25.07 mA cm^2^, and a FF of 85.31%. The performance of the PTAA‐based device falls between those of the two devices, with a PCE of 24.57%, a *V_OC_* of 1.163 V, a *J_SC_* of 25.03 mA cm^2^, and an FF of 84.39%. A certified PCE of 25.34% with a *V_OC_* of 1.182 V, *J_SC_* of 25.07 mA cm^2^, and an FF of 85.50% under reverse scan for a 2PACz‐based device proves the reliability of the photovoltaic performance data obtained in our own laboratory (Figure S3). The negligible differences in light transmittance and conductivity among NiO_x_‐, PTAA‐, and 2PACz‐based substrates would not result in a detectable change in device performance (Figure S4). The difference in photovoltaic performance among NiO_x_‐, PTAA‐, and 2PACz‐based PSCs do not originate from the difference in perovskite bandgap and light absorption level (Figure S5), but from the difference in non‐radiative recombination and charge carrier lifetime (Figure S6 and Table S1), and the trap density level in the perovskite layer (Figure S7), indicated by the significantly higher PL intensity, obviously enhanced average charge carrier lifetime, and lowest trap‐filled limit voltage (*V_TFL_*) for the 2PACz‐based perovskite.

### 2.2 Device Stability Under ISOS‐L‐1I and Solar‐Thermal Cycling Conditions

**FIGURE 2:**
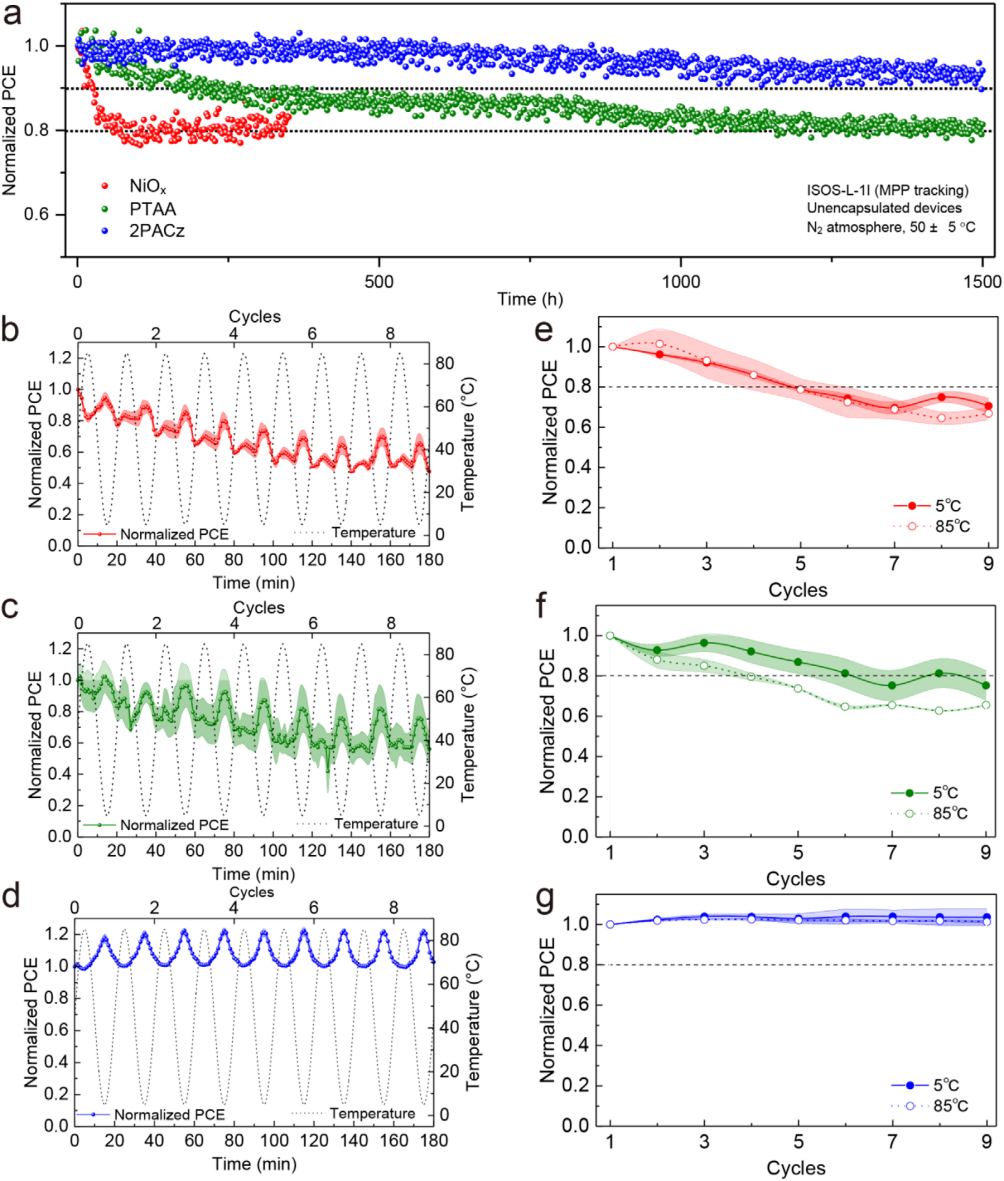
Evolution of the device performance as a function of time under ISOS‐L‐1I and solar‐thermal cycling conditions. (a) Normalized PCE as a function of time under the ISOS‐L‐1I protocol with an interval time of 2 h and a constant 50°C. The NiO_x_ device is monitored until ∼350 h due to its early degradation. Normalized PCE as a function of time under the ISOS‐LT protocol between 5°C and 85°C (dashed line) with each individual cycle duration of 20 min for (b) NiO_x_‐, (c) PTAA‐, and (d) 2PACz‐based devices. The PCE measurements were performed at 1‐min intervals. Normalized PCE taken at 5°C and 85°C in each cycle as a function of thermal cycle numbers for (e) NiO_x_‐, (f) PTAA‐, and (g) 2PACz‐based devices. Values in (**e‐g**) are normalized to the efficiency of the first cycle to illustrate the degradation trend, not for absolute comparison. The shaded areas represent error bars derived from the standard deviation of the respective PCE values of three individual devices.

Due to the limited stability of PSCs, which currently impedes their commercialization, evaluating device stability is necessary [31, 32]. We first measure the device stability over time under the ISOS‐L‐1I measurement protocol with the maximum power point (MPP) tracking model using unencapsulated devices. As shown in Figure 2a, the NiO_x_‐based device exhibits the lowest stability, with only 80% of its initial PCE remaining after a fast decay time of ∼50 h (*T_80_*). In comparison, the PCE of the PTAA‐based device decreases to 80% of the initial PCE after ∼160 h and remains 90% of the initial PCE after ∼1000 h (*T_90_*), indicating a better stability. The 2PACz‐based device exhibits the highest stability with *T_90_* of ∼ 1500 h. Despite the ISOS‐L‐1I protocol offering a stability result of measured devices under a relatively low and constant temperature, the corresponding result is far from the real application of PSCs due to the temperature variation resulting from the day‐night cycles. The actual device temperature can easily reach 65°C under sufficient sunlight conditions, and even reach 85°C under extreme conditions, highlighting the importance of solar‐thermal cycling measurements in the temperature range of 5°C–85°C [33, 34, 35]. The synchrotron‐based *operando* GIWAXS measurements are conducted during device operation with a home‐built setup, as shown in Figure S8, to simultaneously track changes in device performance of PSCs and the structural evolution of the perovskite under rapid solar‐thermal cycling conditions.

The evolution of device performance during *operando* measurements is analyzed to compare the device stability based on NiO_x_, PTAA, and 2PACz EBLs under rapid solar‐thermal cycling (Figure 2b‐d). Overall, the PCE for all devices displays a similar evolution trend with temperature variation, that is, the PCE increases with a temperature fall and decreases with a temperature rise, which is attributed to an enhanced non‐radiative recombination loss resulting from more intense charge carrier motion at high temperatures [33, 36]. For NiO_x_‐based devices, the average PCE undergoes a rapid decay time of 100 min, then tends to stabilize and decrease to 48% of its initial PCE after 180 min of operation. This dramatic degradation in PCE originates from the significant degradation of *J_SC_* (32%), *V_OC_* (21%), and FF (13%), as shown in Figure S9. PTAA‐based devices exhibit similar PCE degradation behavior, that is, the average PCE decreases to 56% of its initial PCE after a fast decay time of 100 min and a fluctuation period of 80 min, originating from the simultaneous degradation of *J_SC_* (32%), *V_OC_* (16%), and FF (3%), as shown in Figure S10. Impressively, the average PCE of 2PACz‐devices shows no degradation after rapid thermal cycling measurements, attributing to the no degradation in *J_SC_*, *V_OC_*, and FF (Figure S11). To further understand the device stability at specific 5°C and 85°C, we extract the PCE at both 5°C and 85°C in each cycle from NiO_x_‐, PTAA‐, and 2PACz‐based devices, as shown in Figure 2e–g, due to the more intense effect of extreme temperature on the device performance. The PCEs at both 5°C and 85°C for the NiO_x_‐based device undergo a similar evolution trend, decreasing to 80% of its initial PCE after 5 thermal cycles, and remaining at ∼70% of its initial PCE after 9 thermal cycles. For the PTAA‐based device, the PCE at 85°C decreases to and fluctuates at 80% of its initial PCE, while the PCE at 5°C decreases to 66% after 9 cycles. The PCE of 2PACz‐based devices always remains the initial value during thermal cycling for both 5°C and 85°C.

### 2.3 Structure Evolution of the Perovskite Crystals

**FIGURE 3:**
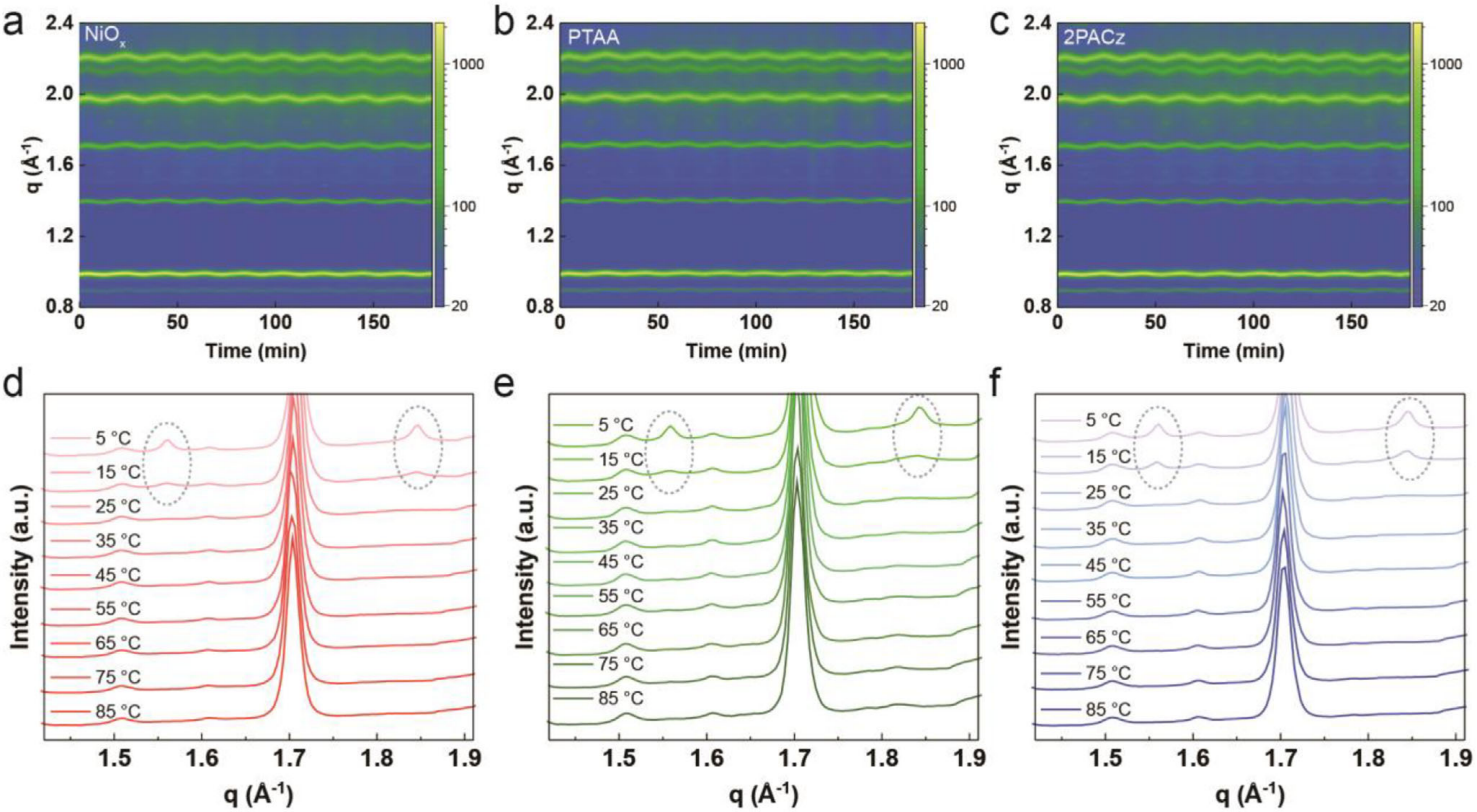
Structure evolution of the perovskite crystals during the *operando* GIWAXS measurements. Temporal GIWAXS evolution plotted by the azimuthal integrated *pseudo*‐XRD data extracted from the 2D GIWAXS data for (a) NiO_x_‐, (b) PTAA‐, and (c) 2PACz‐based devices. Temperature‐dependent *pseudo*‐XRD data of (d) NiO_x_‐, (e) PTAA‐, and (f) 2PACz‐based devices, magnifying at the ***q*** region of 1.42–1.91 Å^−1^, with highlighting of the β‐phases by dashed elliptical circles. The curves are shifted along the y‐axis to avoid curve overlap.

To investigate the degradation behavior of PSCs with different EBLs under rapid thermal cycling conditions, we not only examine the performance evolution but also observe the structural changes during *operando* GIWAXS measurements. To evaluate the effect of different EBLs on the buried side of the perovskite layer, an incidence angle of 0.5° is applied for GIWAXS measurement to ensure the obtaining of structural information from the buried side, as indicated by the ITO substrate signal (Figure S12). Figure 3a–c shows the temporal GIWAXS evolution plotted by the azimuthal integrated *pseudo*‐XRD extracted from 2D GIWAXS data for NiO_x_‐, PTAA‐, and 2PACz‐based devices. In overview, the peaks' periodic oscillation follows the periodic temperature variation in all samples, indicating that the lattice spacing of the perovskite crystals undergoes periodic expansion and shrinkage during the thermal cycling. Obviously, there is no δ‐phase appearing, which is a degradation product of the perovskite. In addition, the perovskite based on NiO_x_, PTAA, and 2PACz EBLs exhibits the same slight decrease in crystallinity, indicated by the same degree of peak intensity and area evolution of the perovskite (001) peak (Figure S13). Interestingly, as indicated in the temporal GIWAXS evolution, during the periodic temperature rise and fall in the thermal cycling measurements, a reversible phase transition from the cubic phase (α‐phase) to the tetragonal phase (β‐phase, ***q*** ≈ 1.56 and 1.85 Å) is found [37]. To clearly see the transition temperature from α‐phase to β‐phase, we plot the selected *pseudo*‐XRD at 5°C–85°C in the first thermal cycle for all samples, as shown in Figure 3d–f. It is obvious that the β‐phase appears when the temperature is lower than room temperature for all samples (Figures S14–S16). This reversible phase transition is driven by the increased lattice stress induced by lattice shrinkage when the temperature is lowered below room temperature (Figure S17). In summary, even though the reversible phase transition and reduced crystallinity are the common reasons for the degradation of PSCs according to reports [29, 30], they are not the main reason for the stability difference among NiO_x_‐, PTAA‐, and 2PACz‐based devices, due to almost the same perovskite structure evolution among them during the thermal cycling measurement.

### 2.4 Strain Evolution Under Solar‐Thermal Cycling Conditions

To better understand the degradation mechanism of PSCs based on NiO_x_, PTAA, and 2PACz EBLs, we gain insight into the strain evolution of the perovskite crystal under solar‐thermal cycling conditions. Angular‐dependent GIWAXS is used to investigate structural information along the vertical direction of samples. For example, surface structural information of thin films can be detected with a small incidence angle, whereas a high incidence angle is required to probe information from the entire sample (Figure S18). To compare the effect of different EBLs on the residual strain along the vertical direction of the perovskite film, we present the magnified *pseudo*‐XRD data of NiO_x_‐, PTAA‐, and 2PACz‐based devices extracted from 2D GIWAXS data measured before the solar‐thermal cycling with different X‐ray incidence angles from 0.2° to 0.5° (Figure S19). Obviously, the perovskite (001) peak position continuously shifts to lower ***q*** values for NiO_x_‐based perovskite when increasing the incidence angles, indicating an enlarged lattice spacing from the film surface to the buried side, which leads to significant residual strain (Figure S20a). The PTAA‐based perovskite exhibits an opposite trend in lattice spacing but also shows a significant residual strain (Figure S20b). The 2PACz‐based perovskite exhibits negligible residual strain, as indicated by the unchanged (001) peak position during variation of the incidence angle (Figure S20c). In other words, the strain relaxation in 2PACz‐based perovskite is responsible for its excellent photovoltaic performance and stability.

**FIGURE 4:**
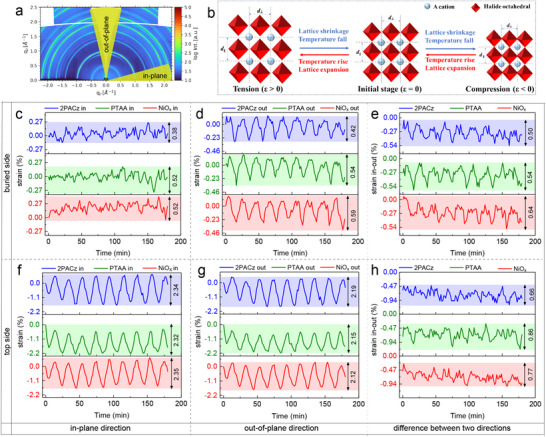
Strain evolution during *operando* GIWAXS measurement under solar‐thermal cycling conditions. (a) Schematic illustration of a cake cut along the in‐plane and out‐of‐plane directions to integrate the *pseudo*‐XRD for strain analysis. (b) Schematic illustration of the lattice expansion and shrinkage of the perovskite crystal under temperature variation. The *d*_∥_ and *d*_⊥_ represent the interplanar spacing parallel to (out‐of‐plane) and perpendicular to (in‐plane) the substrate, respectively. The temporal strain evolution of (c) in‐plane direction, (d) out‐of‐plane direction, and (e) the difference between in‐plane and out‐of‐plane direction, calculated by the peak position evolution of the (001) peak, with an incidence angle of 0.5° of X‐ray. The temporal strain evolution of (f) in‐plane direction, (g) out‐of‐plane direction, and (h) the difference between in‐plane and out‐of‐plane direction, calculated by the peak position evolution of the (001) peak, with an incidence angle of 0.2° of X‐ray.

The strain evolution during *operando* GIWAXS measurement under solar‐thermal cycling conditions is investigated to further understand the device degradation mechanism. Figure 4a schematic illustrates the cake cut along the in‐plane and out‐of‐plane directions in 2D GIWAXS data to integrate the *pseudo*‐XRD for strain analysis during solar‐thermal cycling. Due to the periodic temperature variation during thermal cycling, the lattice spacing undergoes expansion and shrinkage, resulting in different degrees of tension and compression strain along in‐plane and out‐of‐plane directions, as schematically illustrated in Figure 4b. The *d*_∥_ and *d*_⊥_ represent the interplanar spacing parallel to (out‐of‐plane) and perpendicular to (in‐plane) the substrate, respectively. The strain ε is evaluated as follows:

(1) $$\varepsilon =\frac{d_{t}-d_{0}}{d_{0}}$$

where *d*_0_ refers to the initial *d* spacing, and *d_t_* refers to the real‐time *d* spacing at a certain temperature. Similarly, the strain along the in‐plane (ε_*in*_) and out‐of‐plane (ε_*out*_) direction is calculated by Equations S4 and S5, respectively. The temperature variation during solar‐thermal cycling results in the strain along the in‐plane direction fluctuations for all samples (Figure 4c). However, the 2PACz‐based device shows the lowest fluctuation range of 0.38, compared to the same value of 0.52 for both NiO_x_‐ and PTAA‐based devices, indicating fewer lattice expansion and shrinkage for the 2PACz‐based device during thermal cycling, which can be attributed to the strong interaction between 2PACz and perovskite and ITO substrate by the phosphonic acid anchoring group [38]. As shown in Figure 4d, compared with the in‐plane direction, the strain along the out‐of‐plane direction exhibits a larger fluctuation range, regardless of the type of EBLs, due to the decreased interaction between the EBLs and the perovskite along the out‐of‐plane direction (Figure S21). Despite the decrease in the interaction between perovskite and 2PACz along the out‐of‐plane direction, the lattice expansion and shrinkage of perovskite are still limited by 2PACz to a certain extent, resulting in a smaller fluctuation range of 0.42 compared with NiO_x_‐ and PTAA‐based devices (0.59 and 0.54, respectively). Therefore, the difference between the strain along the in‐plane direction and the out‐of‐plane direction (ε_*in*_ − ε_*out*_) for the 2PACz‐based device is lower than that of the NiO_x_‐ and PTAA‐based device, indicating a lower lattice distortion of perovskite during temperature variation for the 2PACz‐based device (Figure 4e). In summary, the 2PACz more efficiently limits the lattice expansion and shrinkage at the buried side of the perovskite layer under temperature variation during thermal cycling, decreasing the lattice distortion.

Apart from the strain evolution at the buried side of the perovskite layer, we also investigate the strain evolution at the surface side of the perovskite during solar‐thermal cycling with a smaller incidence angle of 0.2°. The incidence angle of 0.2° (below the critical angle of perovskites) ensures that the scattering signals from the perovskite surface‐near part are probed, which is verified by the absence of an ITO scattering signal (2.132 Å) in the GIWAXS data (Figure S22). Overall, the strain evolution in both in‐plane (Figure 4f) and out‐of‐plane (Figure 4g) directions changes with temperature variation during solar‐thermal cycling, also for the incidence angle of 0.2°. However, in contrast to the strain evolution measured at an incidence angle of 0.5°, the strain decreases with the rise in temperature and increases with the fall in temperature in the case of the measurement at an incidence angle of 0.2°, indicating that the lattice spacing expands during the fall in temperature and shrinks during the rise in temperature. This abnormal lattice spacing evolution results from the mechanical force formed by the substrate bending induced by the temperature variation [39]. This mechanical force has a much larger effect on the lattice spacing than the thermal expansion and contraction [40, 41], leading to about 4 times the strain observed at 0.2° (∼2.2) compared to 0.5° (∼0.5), which originates from the strong substrate‐induced mechanical clamping, resulting in strain gradient along the vertical direction, and the significant change in perovskite lattice spacing during solar‐thermal cycling. Reported thermal expansion coefficients (CTE) values are approximately 30–80 × 10^−^^6^ K^−^^1^ for hybrid perovskites [42], 11–14 × 10^−^^6^ K^−^^1^ for NiO_x_ [43], and 50–100 × 10^−^^6^ K^−^^1^ for typical organic semiconductor polymers such as PTAA [44]. In contrast, 2PACz forms a self‐assembled monolayer with a thickness of 1–5 nm, and thus its thermal expansion contribution is negligible compared with that of the hundreds‐nanometer‐thick perovskite layer [45]. The extremely small thickness of the EBL layers significantly limits the thermal stress generated during temperature cycling. The strain variation ranges of NiO_x_‐, PTAA‐, and 2PACz‐based devices are at the same level regardless of the in‐plane or out‐of‐plane direction, but the differences in the strain in both directions for the 2PACz‐based device shows the lowest value of 0.66 compared to the 0.77 and 0.86 for NiO_x_‐ and PTAA‐based devices, respectively, indicating a lower lattice distortion of the 2PACz‐based device subjected to temperature variations (Figure 4h). As shown in Figure S23, after a comparative study of the strain evolution between the surface and the buried side of the perovskite layer, it is observed that the strain evolution at the buried side follows the thermal expansion and contraction law, while the strain evolution at the surface is opposite due to the substrate bending inducing mechanical forces. In summary, compared to NiO_x_‐ and PTAA‐based devices, the 2PACz‐based device exhibits a relatively lower lattice distortion on the surface of the perovskite layer due to temperature variation during solar‐thermal cycling conditions, which is explains the longer stability of 2PACz‐based PSCs under thermal cycling.

The smaller lattice distortion observed in the 2PACz‐based devices during solar‐thermal cycling can be explained by considering the interfacial strain state of the perovskite layer. Angular‐dependent GIWAXS measurements before the solar‐thermal cycling (Figure S19) indicate that the perovskite film deposited on 2PACz exhibits minimal residual strain in the studied devices. This lower initial strain state reduces the accumulation of lattice stress during repeated thermal expansion and contraction, thus mitigating structural distortion during thermal cycling [46, 47]. Furthermore, 2PACz forms an ordered, self‐assembled monolayer at the interface. This molecularly ordered interfacial layer may promote a more uniform interfacial stress distribution and suppress localized stress concentrations in the perovskite lattice. These factors contribute to a smaller lattice distortion in 2PACz‐based devices compared to NiO_x_ and PTAA‐based devices.

## 3 Conclusion

This work compares the effects of three typical EBLs (NiO_x_, PTAA, and 2PACz) on the photovoltaic performance and stability of the corresponding PSCs under light soaking and solar‐thermal cycling conditions. The 2PACz‐based devices achieve an impressive PCE of 25.74% (certified at 25.34%), which is significantly higher than 23.35% for NiO_x_‐based devices and 24.57% for PTAA‐based devices, benefiting from a significant improvement in *V_OC_* and a slight increase in FF. The optoelectronic characterization of the perovskite thin films demonstrates that the performance improvement of the 2PACz‐based devices is mainly attributed to the reduced trap density, prolonged charge carrier lifetime, and suppressed non‐radiative recombination. The 2PACz‐based devices exhibit excellent stability under light soaking (>1500 h of *T_90_* lifetime) and rapid solar‐thermal cycling conditions (no PCE degradation). The *operando* GIWAXS measurements reveal that the reversible phase transition and reduced crystallinity are responsible for the degradation of the PSCs; however, strain is the main reason for causing the stability difference among NiO_x_‐, PTAA‐, and 2PACz‐based devices. Comparing the strain evolution between the surface and the buried side of the perovskite layer across all devices reveals that the strain evolution at the buried side follows the thermal expansion and contraction law, whereas the strain evolution at the surface is opposite due to the substrate bending‐inducing mechanical forces. However, compared to NiO_x_‐ and PTAA‐based devices, the 2PACz‐based device exhibits a relatively lower lattice distortion in both the buried side and the surface side of the perovskite layer during solar‐thermal cycling conditions. This finding is a reason for the longer stability of the 2PACz‐based PSCs under thermal cycling conditions. This work provides experimental evidence for the rational selection of EBL materials and new ideas for strain regulation for constructing highly stable perovskite devices for real‐world applications.

## Author Contributions

Xiongzhuo Jiang and Jie Zeng contributed equally to this work. Xiongzhuo Jiang and Jie Zeng conceived the idea, designed the research, performed the experiments, analyzed the data, and wrote the initial manuscript. Kun Sun, Zerui Li, Guangjiu Pan, Simon Alexander Wegener, and Matthias Schwartzkopf performed experiments. Kun Sun, Zerui Li, and Guangjiu Pan analyzed the data and discussed the results. S. Koyiloth Vayalil, Baomin Xu, and Peter Müller‐Buschbaum provided resources. Baomin Xu and Peter Müller‐Buschbaum provided funding, project administration, and supervised the research. All authors discussed the results, contributed to the manuscript writing, provided critical feedback, and approved the final version of the manuscript.

## Funding

This work was supported by funding from the Deutsche Forschungsgemeinschaft (DFG, German Research Foundation) with funding by Germany's Excellence Strategy – EXC 2089/1‐390776260 (e‐conversion) and the International Research Training Group 2022 Alberta/Technical University of Munich International Graduate School for Environmentally Responsible Functional Hybrid Materials (ATUMS), from the Bayerisches Staatsministerium für Wissenschaft und Kunst via TUM.solar in the context of the Bavarian Collaborative Research Project Solar Technologies Go Hybrid (SolTech) and from the Center for NanoScience (CeNS). B. Xu thanks the support from the Guangdong Basic and Applied Basic Research Foundation (2023B1515120031), the Shenzhen Science and Technology Innovation Committee (SGDX20230116091649013), the SUSTech Energy Institute for Carbon Neutrality (High level of special funds, G03034K001), and the technical support from SUSTech Core Research Facilities and the Center for Computational Science and Engineering at SUSTech. X. Jiang and G. Pan acknowledge the financial support from the China Scholarship Council (CSC).

## Conflicts of Interest

The author declares no conflict of interest.

## Supporting information




**Supporting File**: advs75170‐sup‐0001‐SuppMat.pdf.

## Data Availability

The data that support the findings of this study are available from the following public repository: https://doi.org/10.14459/2026mp1848878.
