# Supplementary material for: Degradation Mechanisms Associated with Electron‐Blocking Layers in Inverted Perovskite Solar Cells
Source: Adv Sci (Weinh). 2026 Apr 7;13(36):e75170. doi: 10.1002/advs.75170 (PMC13317564; doi:10.1002/advs.75170)
Supplement: Supplementary file 1 — Supporting File: advs75170‐sup‐0001‐SuppMat.pdf. [file ADVS-13-e75170-s001.pdf]

Supporting Information

Degradation Mechanisms Associated with Electron-Blocking Layers in Inverted Perovskite Solar Cells

Xiongzhuo Jiang, Jie Zeng, Kun Su, Simon Alexander Wegener, Zerui Li, Guangjiu Pan, Sarathlal Koyiloth Vayalil, Matthias Schwartzkopf, Baomin Xu, Peter Müller-Buschbaum**

X. Jiang, J. Zeng, B. Xu
Department of Materials Science and Engineering, Southern University of Science and Technology, Shenzhen 518055, China
E-mail: xubm@sustech.edu.cn

X. Jiang, K. Sun, S.A. Wegener, Z. Li, G. Pan, P. Müller-Buschbaum
Technical University of Munich, TUM School of Natural Sciences, Department of Physics, Chair for Functional Materials, James-Franck-Str. 1, 85748 Garching, Germany
E-mail: muellerb@ph.tum.de

X. Jiang
Shenzhen BTR New Energy Technology Institute Co., Ltd., Shenzhen, Guangdong 518118, China

K. Sun
Helmholtz-Zentrum Berlin für Materialien und Energie GmbH, Department Perovskite Tandem Solar Cells, Kekuléstraße 5, 12489 Berlin, Germany

S. Koyiloth Vayalil, M. Schwartzkopf
Deutsches Elektronen-Synchrotron DESY, Notkestr. 85, 22607 Hamburg, Germany

S. Koyiloth Vayalil
Applied Sciences Cluster, University of Petroleum and Energy Studies UPES, Dehradun, Uttarakhand, 248007, India

X. Jiang and J. Zeng contributed equally to this work.

Experimental Section

Materials

Lead (II) iodide (PbI_2, 99.99%, trace metal basis) and [2-(9H-Carbazol-9-yl)ethyl]phosphonic acid (2PACz, >98%) were purchased from Tokyo Chemical Industry (TCI). Formamidinium iodide (FAI, >99.99%) was purchased from GreatCell Solar Materials. Cesium iodide (CsI, 99.99%), methylammonium iodide (MAI, 99.5%), methylammonium chloride (MACl, 99.5%), poly[bis(4-phenyl)(2,4,6-trimethylphenyl)amine (PTAA), bathocuproine (BCP, 99.9%), C_{60} (99.9%), molybdenum (VI) oxide (MoO_3), and piperazinium iodide (PI, 99.5%) were bought from Xi'an YuriSolar. Silver (Ag, 99.999%) was purchased from ZhongNuo Advanced Material (Beijing) Technology. N, N-dimethylformamide (DMF, 99.5%), dimethyl sulfoxide (DMSO, 99.5%), chlorobenzene (CB, 99.5%), and isopropanol (IPA, 99.5%) were purchased from J&K. Unless stated otherwise, all solvents were used without further purification.

Perovskite precursor preparation

For the composition $Cs_{0.05}FA_{0.85}MA_{0.1}PbI_3$, 1.63 M perovskite precursor solution was prepared by mixing aqueously synthesized $FAPbI_3$, $MAPbI_3$, $CsPbI_3$ microcrystals as in our previous work[1] with the molar ratio of 0.85: 0.1: 0.05 and an additional 10 mol% MACl in mixed solvents of DMF: DMSO= 4:1(vol/vol) and then stirred at room temperature for 2 h before using.

Device fabrication

Indium tin oxide (ITO) glass (1.5 cm × 1.5 cm and 2.5 cm × 2.5 cm, 9 Ω/sq, Advanced Election Technology) was used as the substrate. The substrates were cleaned by sonication with detergent (Decon 90), deionized water, and ethyl alcohol for 30 min each. Then, the substrates were dried with N_2 blowing and exposed to UV-ozone treatment for 30 min before use.

Deposition of EBLs: For the NiO_x layer, freshly prepared NiO_x nanoparticles ink (10 mg ml^{-1} dispersed in deionized water) was spin-coated onto the cleaned ITO substrate at 3000 rpm for 40 s, then annealed at 120 °C for 10 min in ambient air. The PTAA (2 mg/mL in chlorobenzene) layer was deposited on the ITO substrate by spin-coating (4000 rpm for 30 s), followed by thermal annealing at 100 °C for 10 min. The 2PACz layer was fabricated by spin-coating the 2PACz solution (0.5 mg/mL in IPA) onto the ITO substrate at 3000 rpm for 30 s and then annealed at 100 °C for 10 min. A washing spin-coating step (with IPA) was conducted at 3000 rpm for 20 s to remove the excess 2PACz molecules.

Deposition of perovskite absorber: 70 μL of the precursor solution was spread onto the HTM substrate, and spin-coated using a two-stage procedure (1000 rpm for 10 s and 5000 rpm for 30 s), with 180 μL of CB as the antisolvent dripped at 12 s during the second stage. The as-prepared perovskite films were annealed at 100 °C for 30 min.

After cooling to room temperature, 60 μL of PI solution (0.5 mg mL-1in IPA: DMF = 200:1 (vol/vol)) as a passivation layer was spin-coated onto the perovskite layer at 5000 rpm for 30 s, followed by annealing at 100 °C for 5 min. Finally, the whole device was transferred to a vacuum chamber under a base pressure of 2×10^{-5} torr. As a stack of 40 nm C_{60}, 8 nm BCP, and 100 nm Ag was sequentially thermal evaporated with a shadow mask.

***Operando* GIWAXS measurements under solar-thermal cycling conditions**

The *operando* GIWAXS data under solar-thermal cycling were recorded by a Lambda 9M detector (X-Spectrum) with a beam energy of 11.87 keV at beamline P03 at PETRA III synchrotron (DESY, Hamburg). The data was collected with a sample-to-detector distance (SDD) of 243 mm and an exposure time of 1s per frame. The SDD was calibrated with LaB_6 and CeO_2 powders with the DPDAK package and further calibrated with the ITO peak (2.132 $Å^{-1}$).[2] To probe the structure information from different depths of the perovskite layer, a varied set of incidence angles from 0.2-0.5° (step of 0.05°) was applied. The data reduction, including transformation to *q*-space, detector absorption, solid angle, and linecuts, was processed by using the Python tool INSIGHT.[3] The setup was connected with a cooling-water system (Julabo) to exclude external heat-induced degradation, as in the previous study.[4-5] In particular, a home-built sample holder with a Peltier element is integrated in the sample holder, enabling rapid heating and cooling in the range of 5 °C to 85 °C with each thermal cycling duration of 20 min. During the *operando* measurements, the current density-voltage (*J-V*) curves were measured within -0.2 to 1.2 V under AM 1.5 G illumination (100 W/m^2) by a Xenon short-arc lamp (PE150AF, Excelitas Technologies), with an interval time of 1 min.

Stability tests under the ISOS-L-1I protocol

For stability assessment, we fabricated inverted perovskite solar cells (PSCs) with an architecture of ITO/EBLs/perovskite/PI/C_{60}/BCP/Ag. Operational stability was evaluated on a commercial multichannel stability test system (Wuhan91PVKSolar). The encapsulated devices were tested at the MPP under one-sun illumination in N_2 atmosphere. The illumination was achieved by a one-sun-equivalent white-light LED, and the light intensity was calibrated to achieve the same J_{SC} from the *J-V* results. The temperature of the chamber was maintained at

50 ± 5 °C. The bias at the MPP was calculated and applied automatically. The initial Vmax of MPP was obtained by *J-V* sweep and then updated by the perturb-and-observe algorithm. *J-V* sweep was conducted per 2 h.

Materials characterizations

A Perkin Elmer Lambda 35 was used to measure the UV-Vis spectra with a scan speed of 480 nm min^{-1}. Steady-state PL and time-resolved PL spectra were recorded by the Edinburgh FLS 1000 spectrometer using an excitation wavelength of 450 nm. The sheet resistance was measured by a four-point probe setup with a Keithley 2400 source meter.

Devices characterizations

All *J-V* curves of devices with a small effective area were characterized in an N_2-filled glovebox using a Keithley 2400 source meter and an Enlitech Solar Simulator SS-F7-3A, which provided simulated AM 1.5G illumination (100 mW cm^{-2}). The spectrum calibration was performed using a NIST-certified monocrystalline Si solar cell (Newport 532 ISO1599). The *J-V* curves were obtained from a forward scan (from -0.1 V to 1.2 V) and a reverse scan (from 1.2 V to -0.1 V) with a scanning step of 0.01 V and a delay time of 10 ms. The active area of solar cells was defined by the aperture of the metal mask (0.068 cm^2). The space-charge-limited current (SCLC) was measured with the hole-only device architecture of ITO/EBLs/perovskite/PTAA/Ag within the range of 0-5 V.

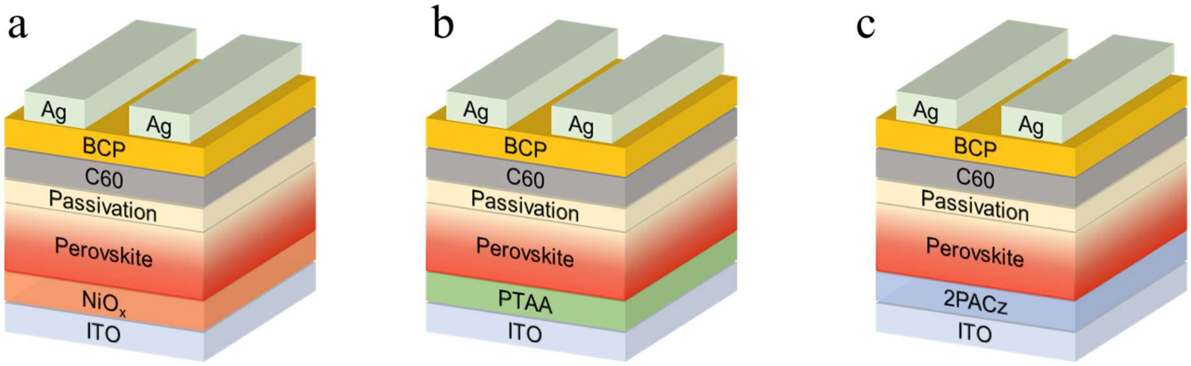

Figure S1 Device architecture of PSCs based on (a) NiO_x, (b) PTAA, and (c) 2PACz used in this study.

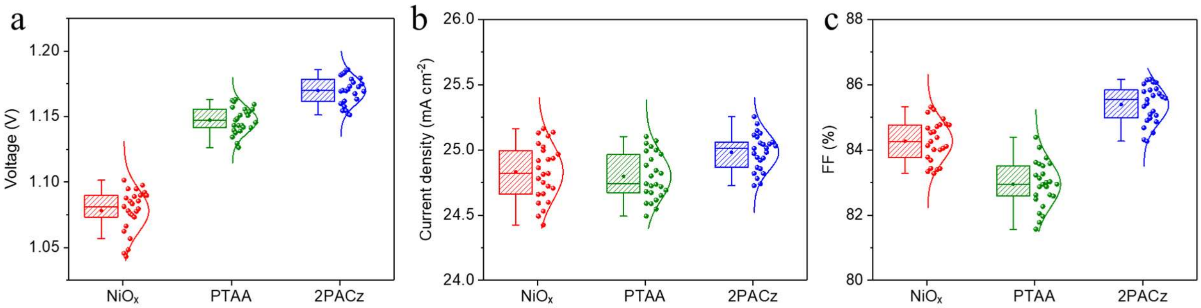

Figure S2 Box plots of (a) V_{OC}, (b) J_{SC}, and (c) FF for PSCs based on NiO_x, PTAA, and 2PACz EBLs.

SiMIT Report No. 23TR112202

Sample Information	
Sample Type	Inverted Perovskite Photovoltaic Cell
Serial No.	Z-11#
Lab Internal No.	23112201-2#
Measurement Item	I-V characteristic
Measurement Environment	24.1±2.0°C,42.4±5.0%R.H

Measurement of I-V characteristic	
**Reference cell**	PVM 1121
**Reference cell Type**	mono-Si, WPVS, calibrated by NREL (Certificate No. ISO 2075)
**Calibration Value/Date of Calibration for Reference cell**	144.53mA/ Feb. 2023
**Measurement Conditions**	Standard Test Condition (STC):<br>Spectral Distribution: AM1.5 according to IEC 60904-3 Ed.3,<br>Irradiance: 1000±50W/m², Temperature: 25±2°C
**Measurement Equipment/ Date of Calibration**	AAA Steady State Solar Simulator (YSS-T155-2M) / July.2023<br>IV test system (ADCMT 6246) / June. 2023<br>SR Measurement system (CEP-25ML-CAS) / April.2023<br>Measuring Microscope (MF-B2017C) / July.2023
**Measurement Method**	I-V measurement:<br>Logarithmic sweep in both directions (Voc to Isc and Isc to Voc) during one flash based on IEC 60904-1:2020.<br>Spectral Mismatch factor was calculated according to IEC 60904-7 and I-V correction according to IEC 60891.
**Measurement Uncertainty**	Area: 1.0%(k=2); Isc: 2.0%(k=2); Voc: 1.0%(k=2);<br>Pmax: 2.4%(k=2); Eff:2.5%(k-2)

2 / 3

SiMIT Report No. 23TR112202

====Measurement Results ====

	Forward Scan (Isc to Voc)	Reverse Scan (Voc to Isc)
Area	6.87 mm²	
Isc	1.722 mA	1.722 mA
Voc	1.182 V	1.182 V
Pmax	1.720 mW	1.741 mW
Ipm	1.647 mA	1.648 mA
Vpm	1.045 V	1.057 V
FF	84.56 %	85.50 %
Eff	25.04 %	25.34 %

- Spectral Mismatch Factor: SMM=0.9923.
- Designated illumination area defined by a thin mask was measured by measuring microscope.
- Test results listed in this measurement report refer exclusively to the mentioned measured sample.
- The results apply only at the time of the test, and do not imply future performance.

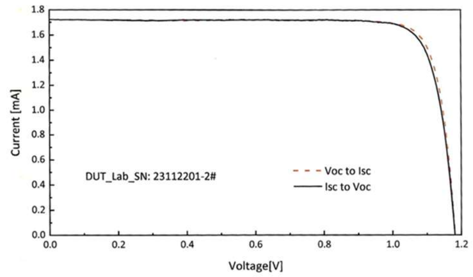

Fig.1 I-V curves of the measured sample

-------**End of Report**-------

3 / 3

Figure S3 Certified photovoltaic parameters and *J-V* curve of 2PACz-based device.

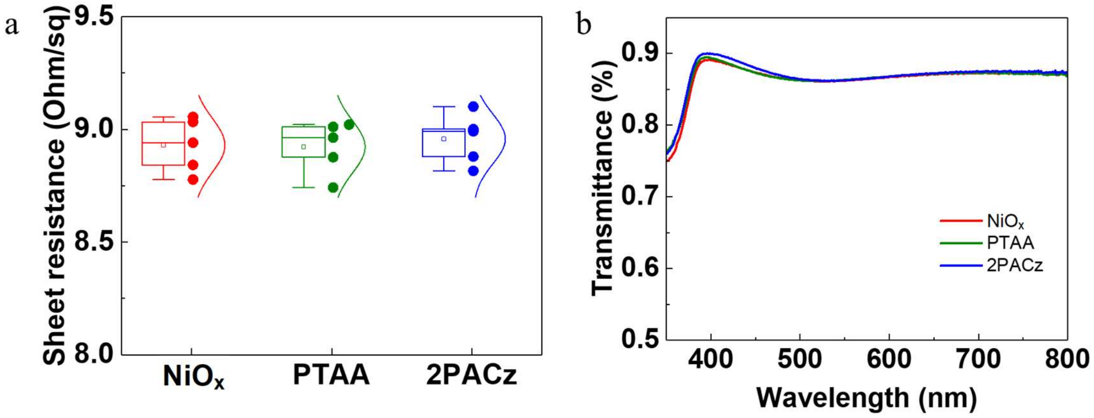

Figure S4. The (a) sheet resistance and (b) UV-Vis transmittance spectra for NiO_x-, PTAA-, and 2PACz-based substrate.

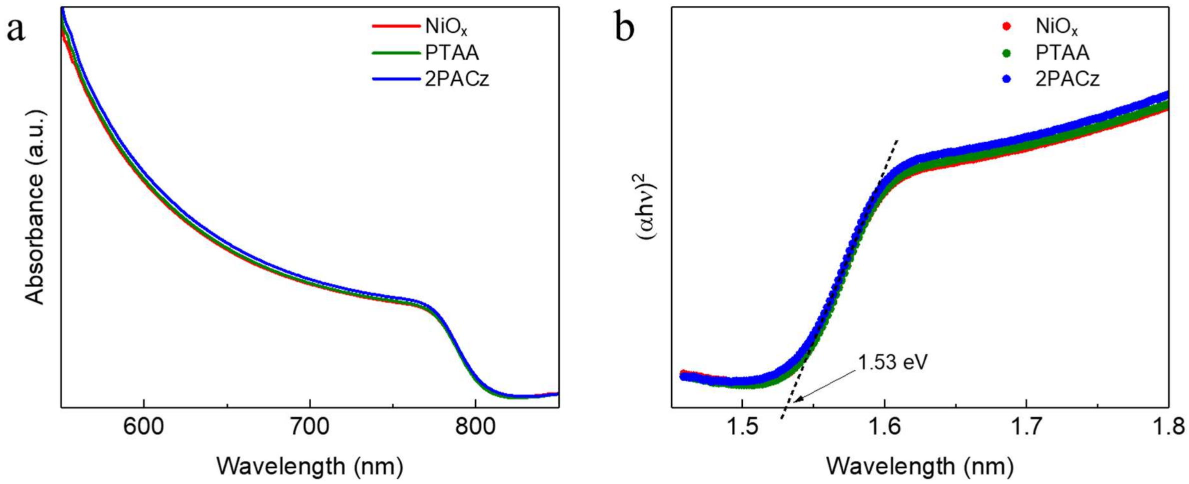

Figure S5 (a) UV-Vis spectra and (b) corresponding Tauc plot of perovskite thin films based on NiO_x, PTAA, and 2PACz.

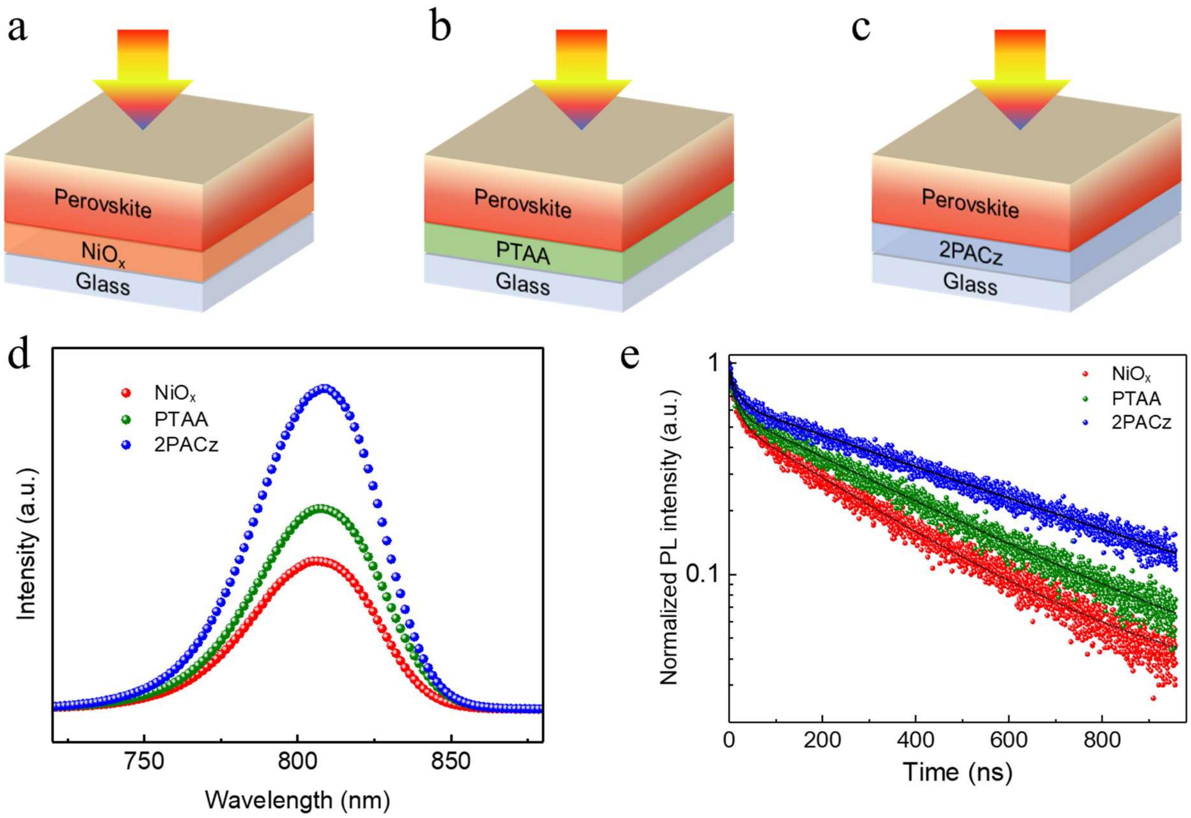

Figure S6 Sample structure for PL and TRPL measurements of perovskite films deposited on top of (a) NiO_x, (b) PTAA, and (c) 2PACz with an excitation direction from the perovskite film side. (d) PL spectra and (e) TRPL spectra of perovskite thin films based on NiO_x, PTAA, and 2PACz.

Table S1. Fit parameters of the bi-exponential fits for analysis of the time-resolved PL spectra.

Perovskite film	A_1	τ_1 ns	A_2	τ_1 ns	$\tau_{average}$ ns
NiO_x-based	0.39	15.7	0.51	301.3	289.8
PTAA-based	0.34	15.6	0.57	394.4	385.7
2PACz-based	0.28	18.1	0.64	567.1	559.5

Note S1: The TRPL decay is fitted by a bi-exponential equation[6-7]:

$$y = A_1 \exp\left(-\frac{t}{\tau_1}\right) + A_2 \exp\left(-\frac{t}{\tau_2}\right) + y_0 \qquad (S1)$$

where parameters A_1 and A_2 are the amplitude fraction for each decay component, τ_1 and τ_2 represent the time constant of the two types of decay, and y_0 is a constant. The average lifetime ($\tau_{average}$) can be calculated with the **Equation S2** :[8]

$$\tau_{average} = \frac{A_1\tau_1^2 + A_2\tau_2^2}{A_1\tau_1 + \tau_2\tau_2} \qquad (S2)$$

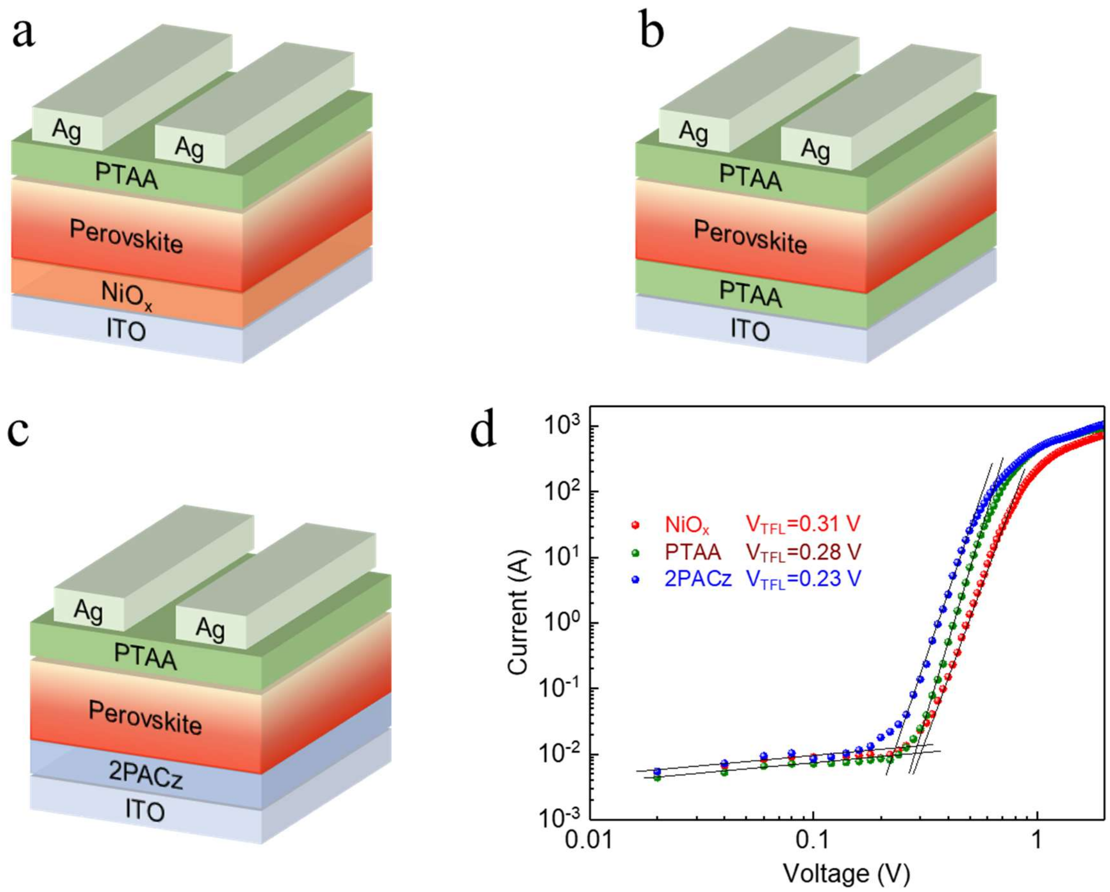

Figure S7 Hole-only devices based on (a) NiO_x, (b) PTAA, and (c) 2PACz EBLs for space-charge-limited current measurement.

Note S2: The trap density (N_t) can be calculated according to the **Equation S3**:[8-10]

$$N_t = \frac{2\varepsilon\varepsilon_0 V_{TFL}}{qL^2} \tag{S3}$$

where V_{TFL} stands for the onset voltage of the trap-filled limited region, L is the thickness of the perovskite film, q denotes the elementary charge, and ε and ε_0 represent the dielectric constant of the perovskite and the vacuum, respectively. The 2PACz-based device exhibits the lowest V_{TFL} of 0.23 V, compared with NiO_x- and PTAA-based devices (0.31 V and 0.28 V). Accordingly, a reduced trap density of 2.10×10^{15} cm^{-3} is achieved for a 2PACz-based device, which is lower than NiO_x- and PTAA-based devices of 2.86×10^{15} cm^{-3} and 2.58×10^{15} cm^{-3}, respectively.

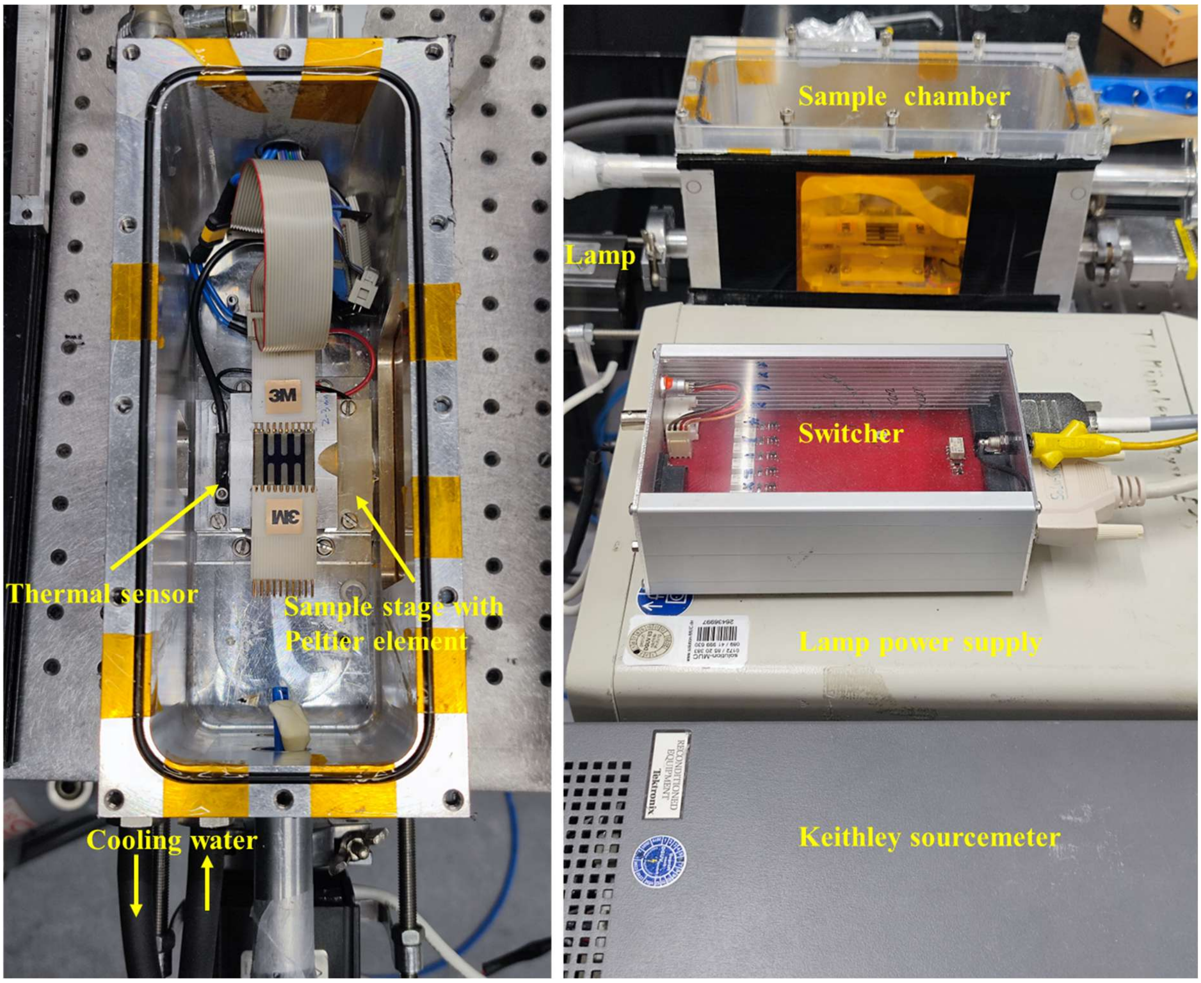

Figure S8 Experimental setup for solar-thermal cycling measurements and *operando* GIWAXS measurements at DESY, P03 beamline.

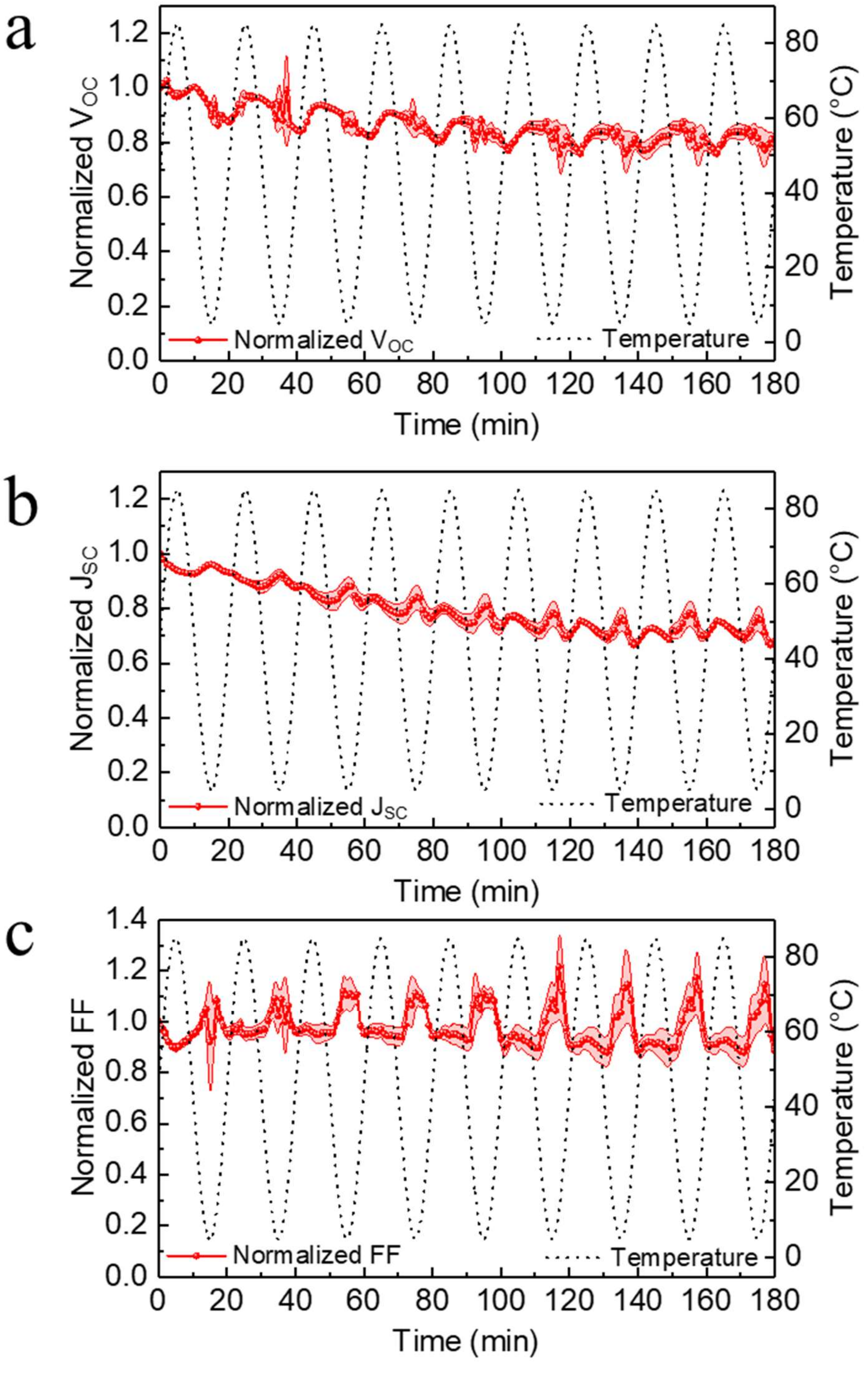

Figure S9 Evolution of (a) normalized V_{OC}, (b) normalized J_{SC}, and (c) normalized FF for NiO_x-based devices under rapid thermal cycling in the temperature range of 5-85 °C.

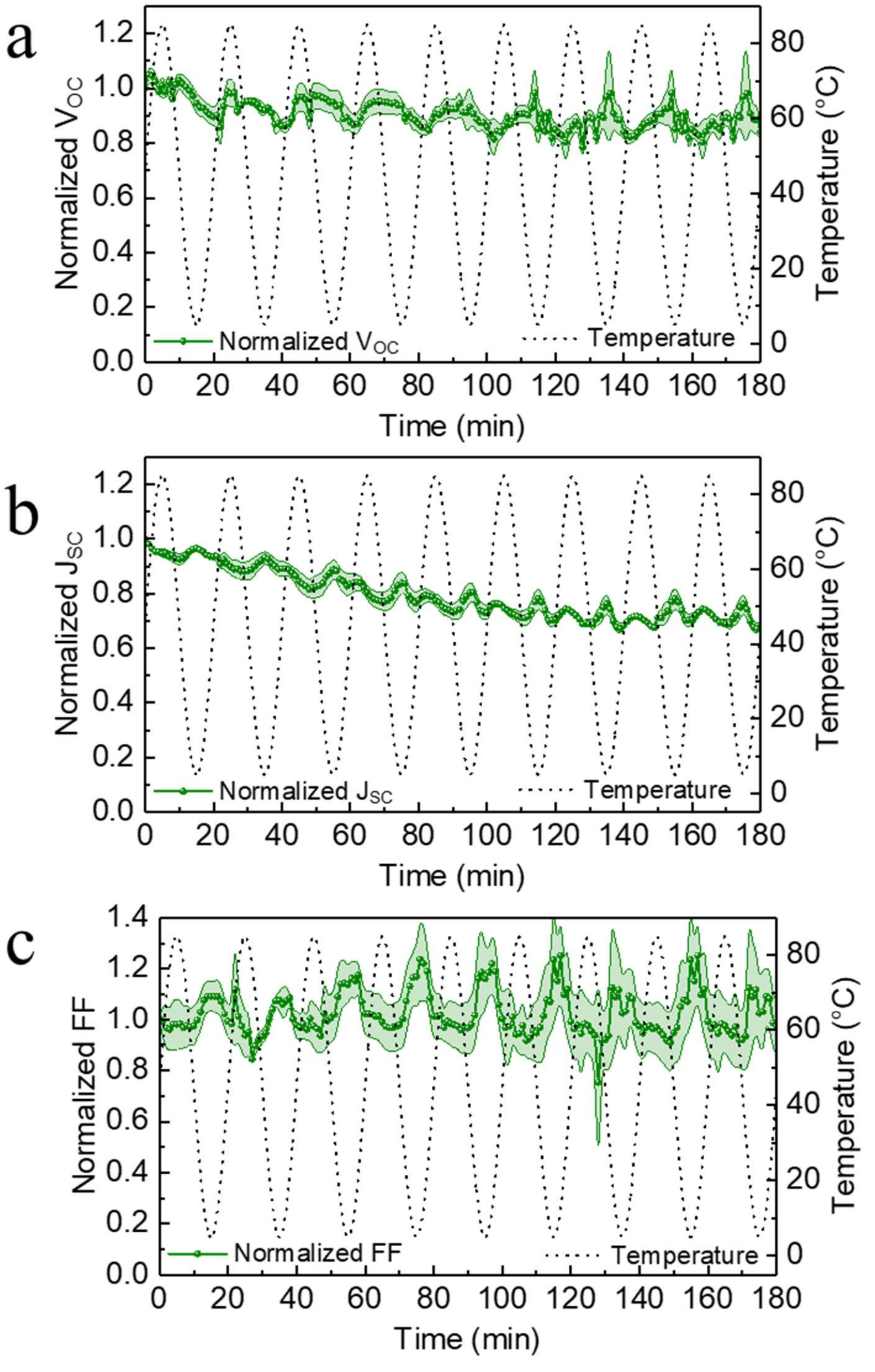

Figure S10 Evolution of (a) normalized V_{OC}, (b) normalized J_{SC}, and (c) normalized FF for PTAA-based devices under rapid thermal cycling in the temperature range of 5-85 °C.

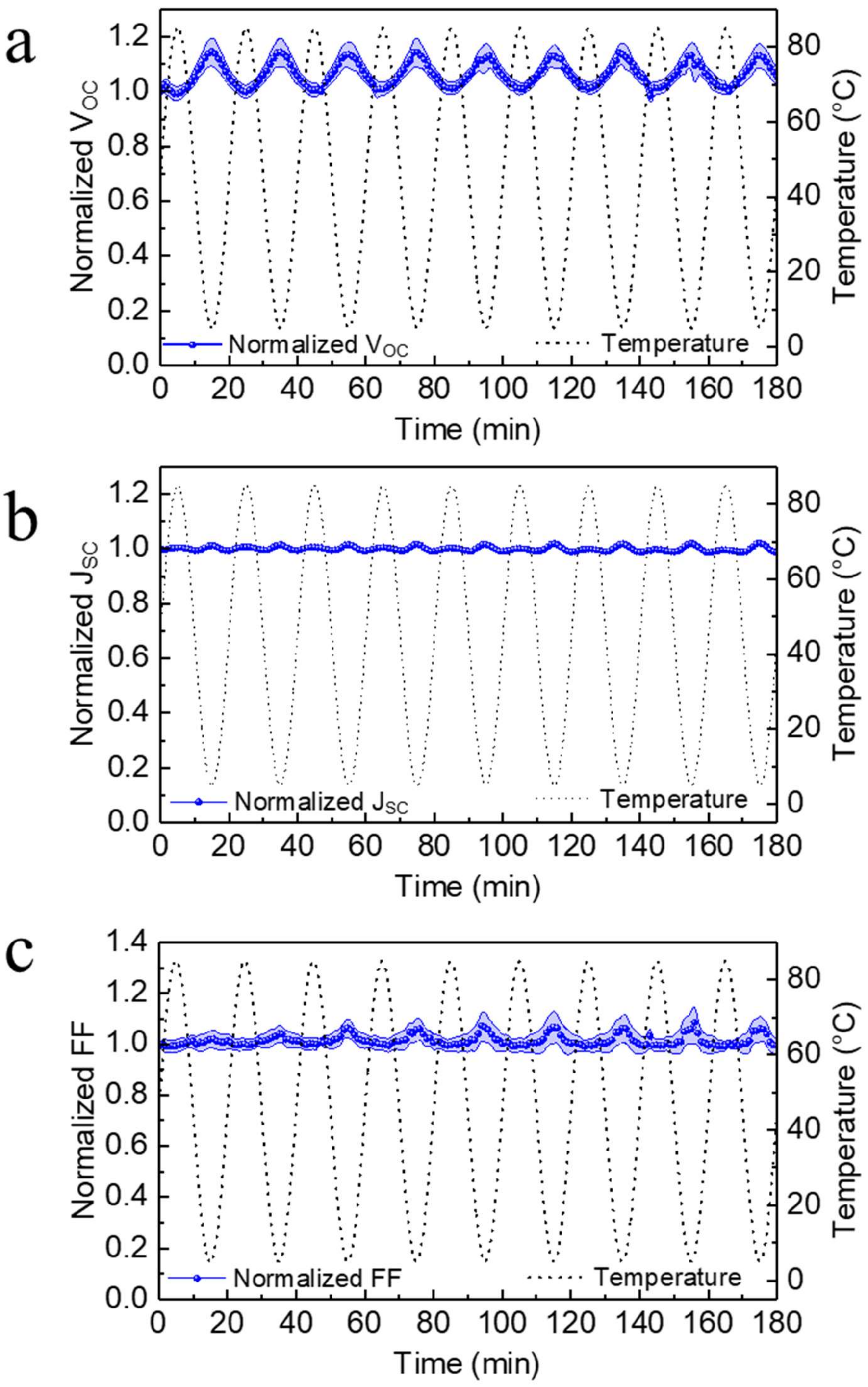

Figure S11 Evolution of (a) normalized V_{OC}, (b) normalized J_{SC}, and (c) normalized FF for 2PACz-based devices under rapid thermal cycling in the temperature range of 5-85 °C.

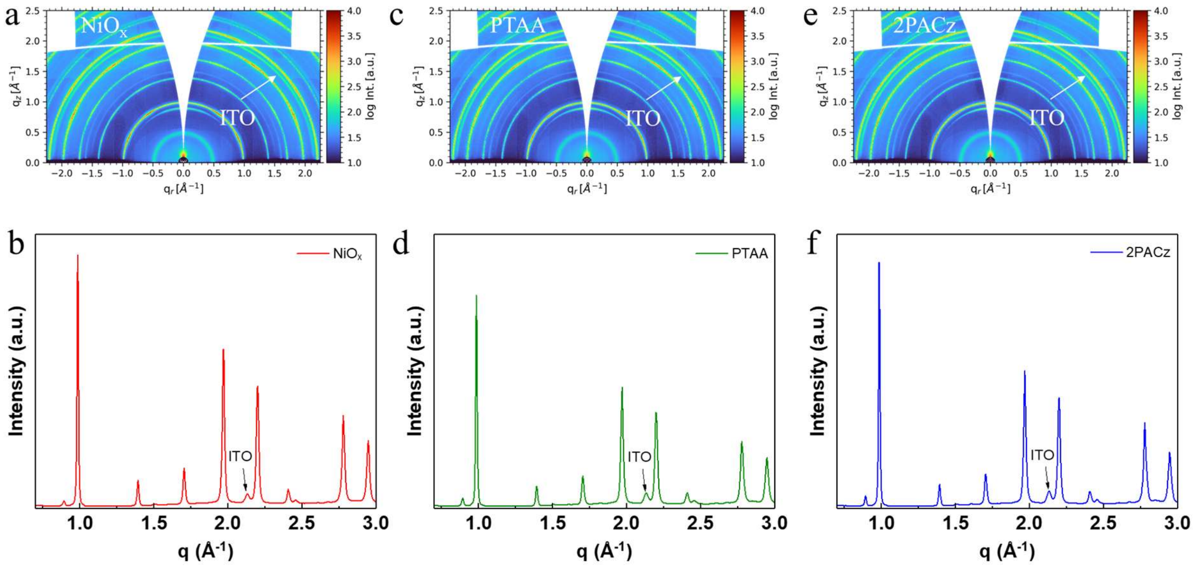

Figure S12 2D GIWAXS data of (a) NiO_x-, (c) PTAA-, and (e) 2PACz-based devices with an incidence angle of 0.5°. The correlation pseudo-XRD of (b) NiO_x-, (d) PTAA-, and (f) 2PACz-based devices. The ITO scattering ring and peak are indicated by an arrow.

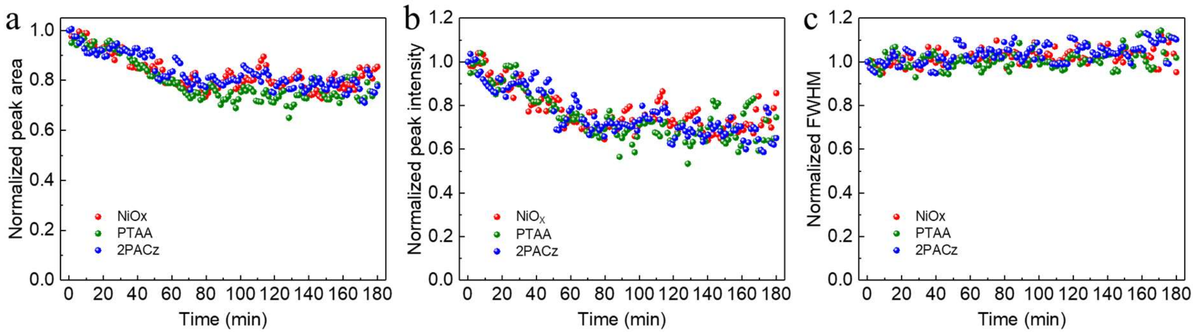

Figure S13 Evolution of normalized (a) peak area, (b) peak intensity, and (c) full width at half maximum (FWHM) of the perovskite (001) peak for NiO_x-, PTAA-, and 2PACz-based device.

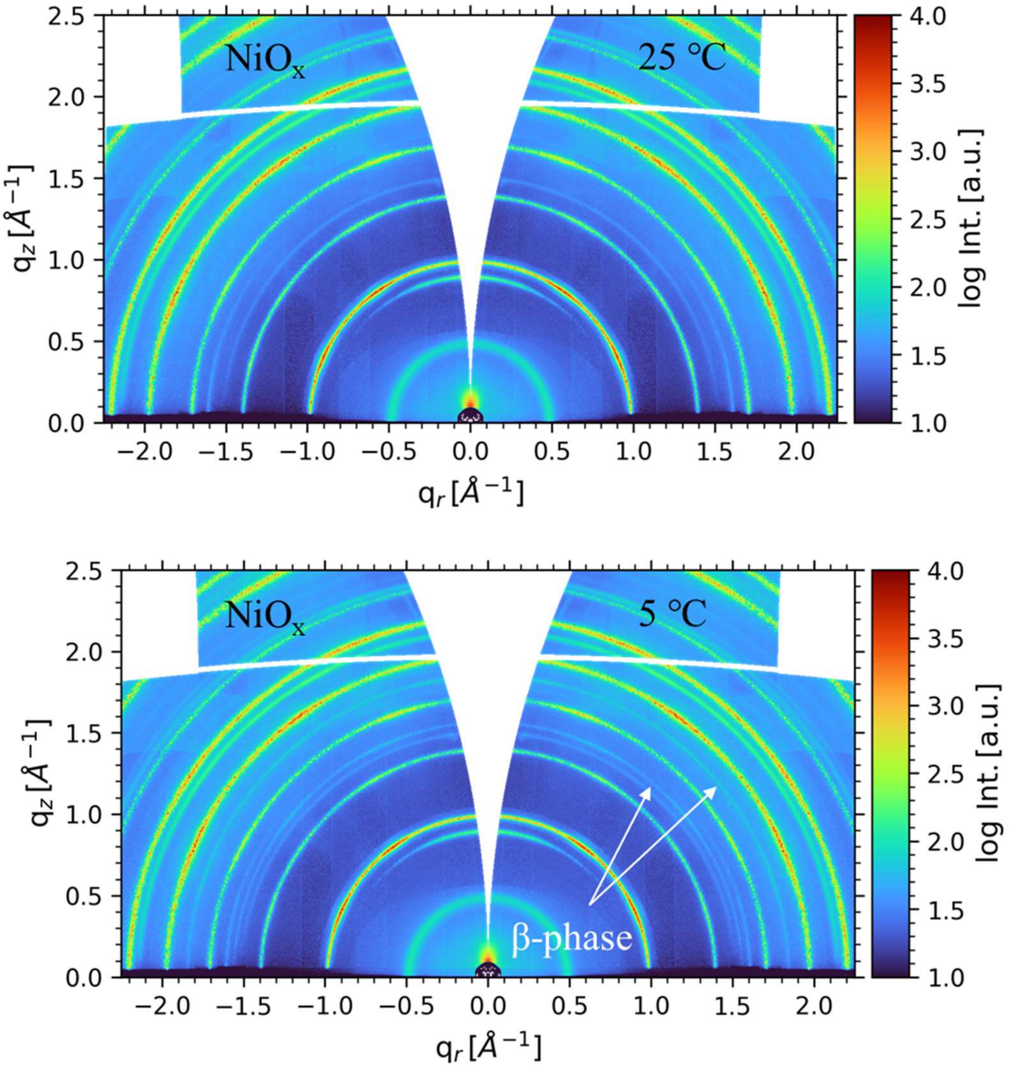

Figure S14 2D GIWAXS data of the NiO_x-based device at 25 °C and 5 °C selected from the first thermal cycle, showing the β-phase appearing at low temperatures as indicated with arrows.

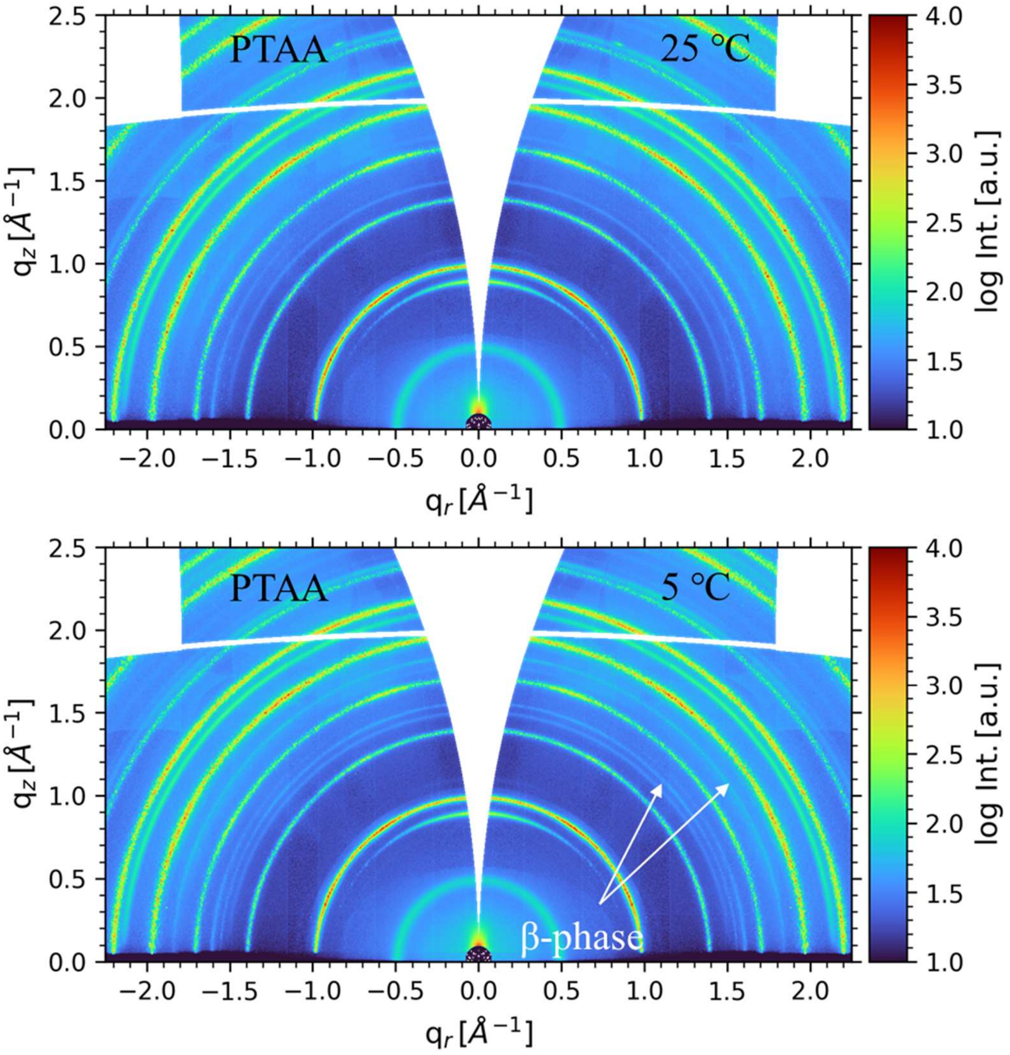

Figure S15 2D GIWAXS data of the PTAA-based device at 25 °C and 5 °C selected from the first thermal cycle, showing the β-phase appearing at low temperatures as indicated with arrows.

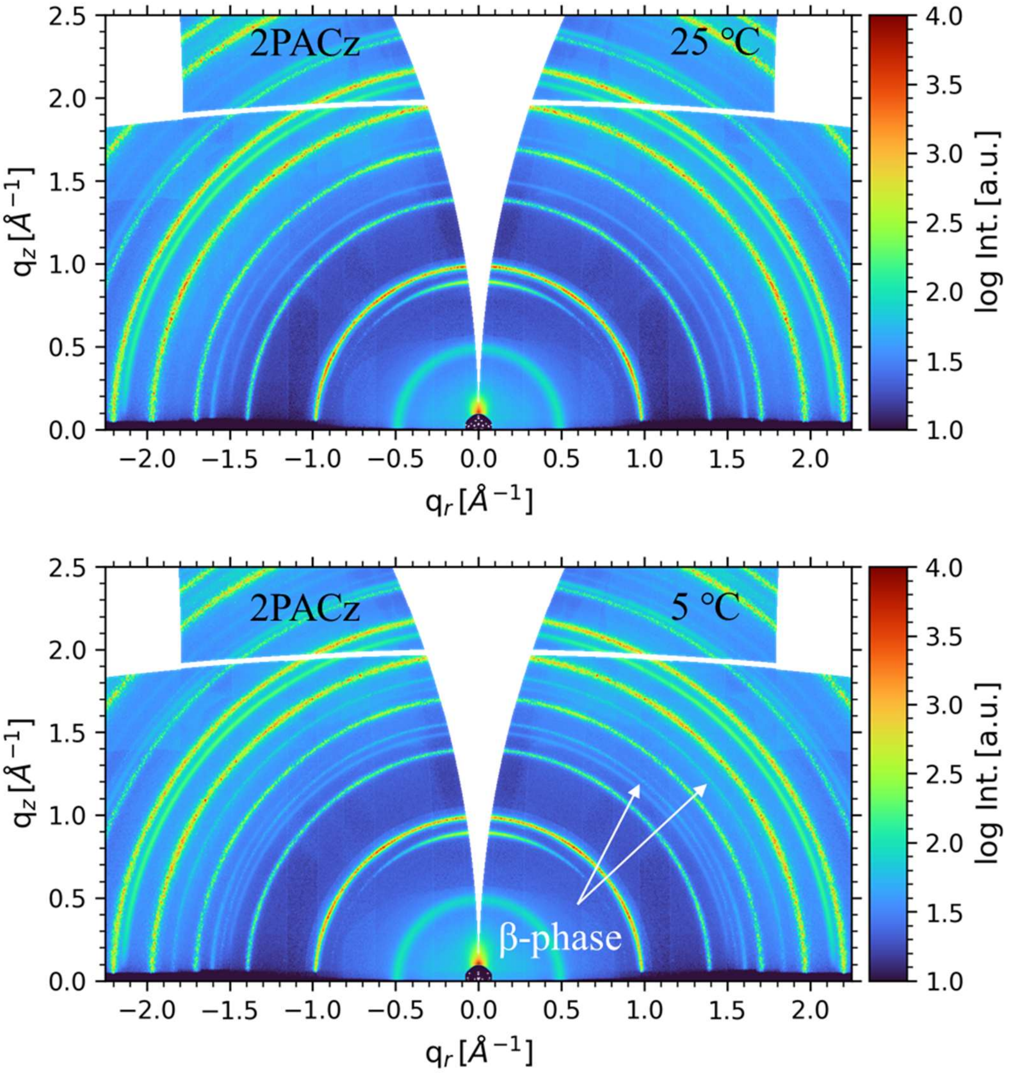

Figure S16 2D GIWAXS data of the 2PACz-based device at 25 °C and 5 °C selected from the first thermal cycle, showing the β-phase appearing at low temperatures as indicated with arrows.

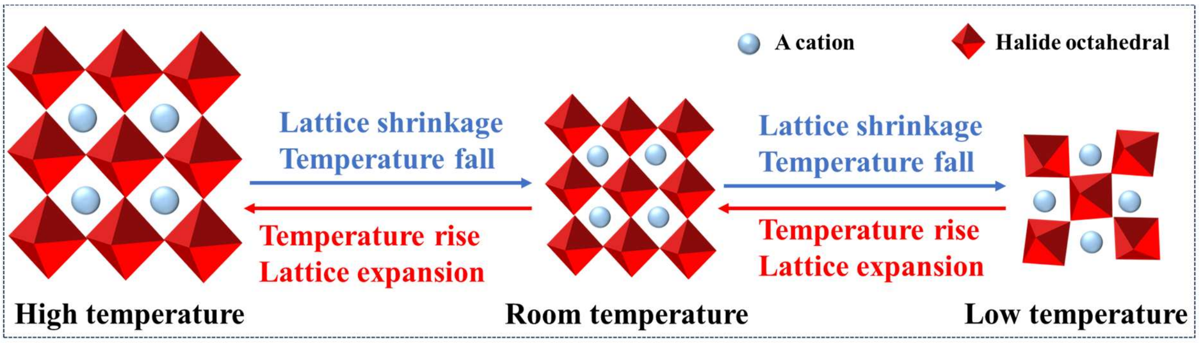

Figure S17 Schematic illustration of perovskite crystal evolution under rapid thermal cycling, showing lattice expansion and shrinkage during temperature rise and fall, respectively, and the phase transition at low temperatures.

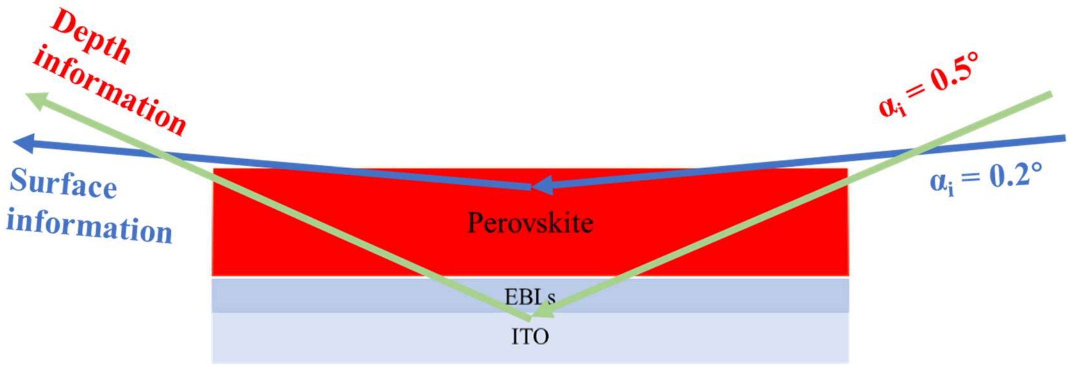

Figure S18 Schematic illustration of angular-dependent GIWAXS measurements with samples of different incidence angles (α_i) at 0.2° and 0.5°.

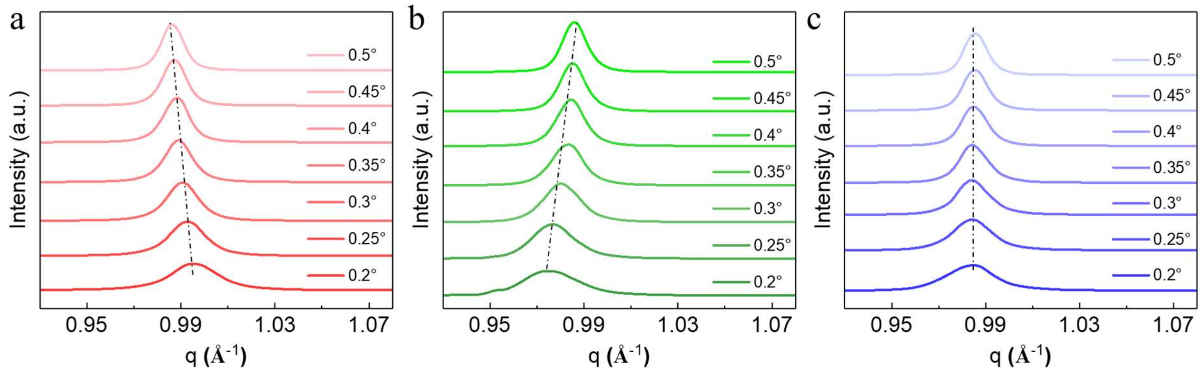

Figure S19 Magnified pseudo-XRD data of (a) NiO_x-, (b) PTAA-, and (c) 2PACz-based devices extracted from 2D GIWAXS data measured before the solar-thermal cycling with different X-ray incidence angles from 0.2-0.5°.

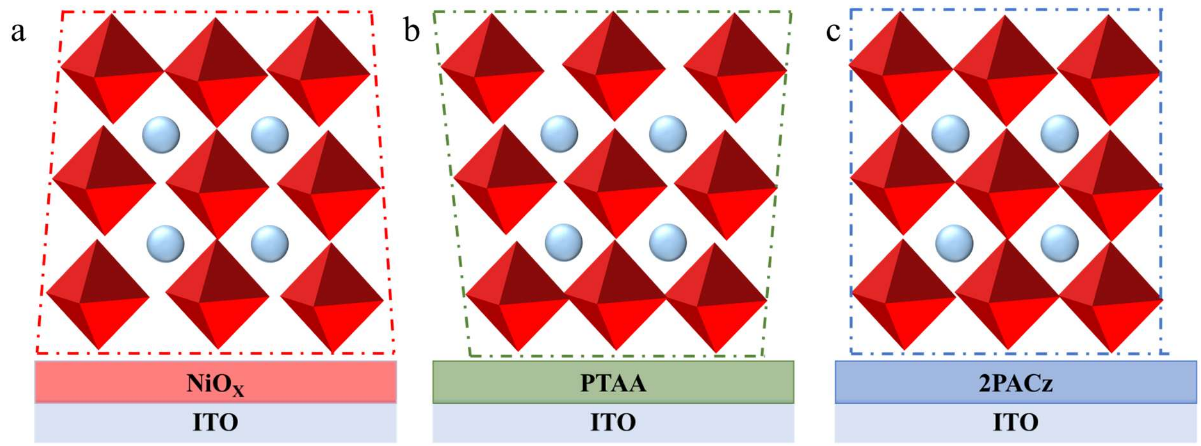

Figure S20 The schematic diagram of residual strain distribution along the vertical direction in perovskite film based on (a) NiO_x, (b) PTAA, and (c) 2PACz devices.

Note S3: The strain along the in-plane direction (ε_{in}) is calculated by **Equation S4**:

$$\varepsilon_{in} = \frac{d_{\perp,t} - d_{\perp,0}}{d_{\perp,0}} \quad (S4)$$

where $d_{\perp,0}$ refers to the initial d spacing, $d_{\perp,t}$ refers to real-time d spacing at a certain temperature. Similarly, the strain along the in-plane direction (ε_{out}) is calculated by **Equation S5**:

$$\varepsilon_{in} = \frac{d_{\parallel,t} - d_{\parallel,0}}{d_{\parallel,0}} \quad (S5)$$

where $d_{\parallel,0}$ refers to the initial d spacing, $d_{\parallel,t}$ refers to real-time d spacing at a certain temperature.

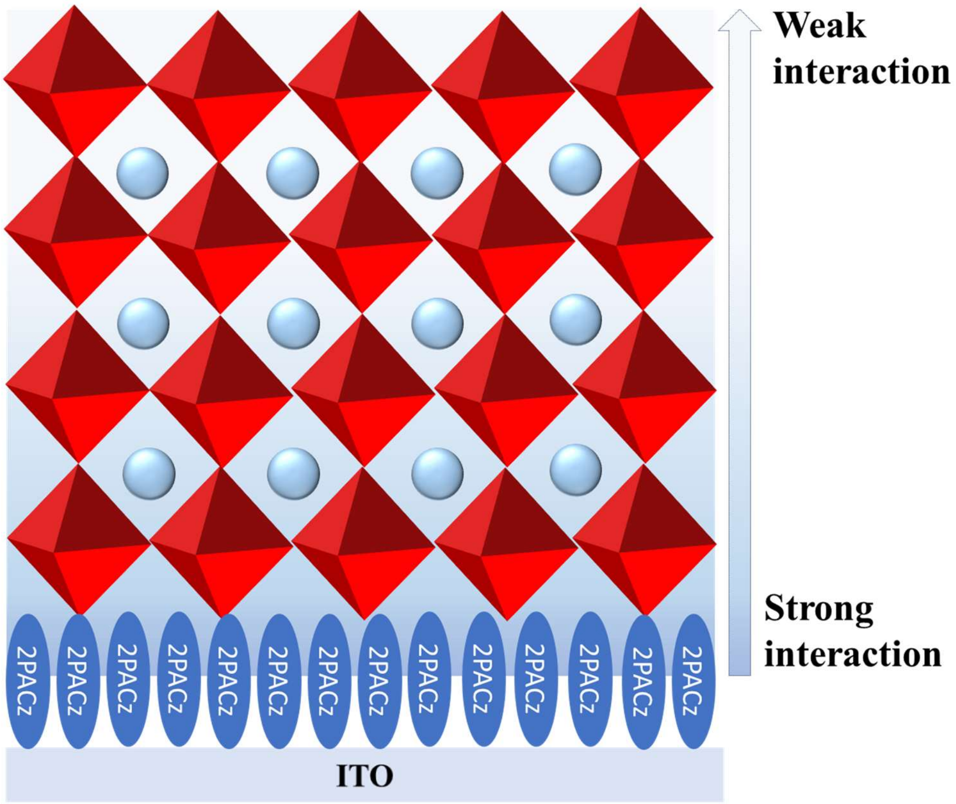

Figure S21 Schematic diagram of the interaction between EBLs (e.g., 2PACz) and the perovskite layer.

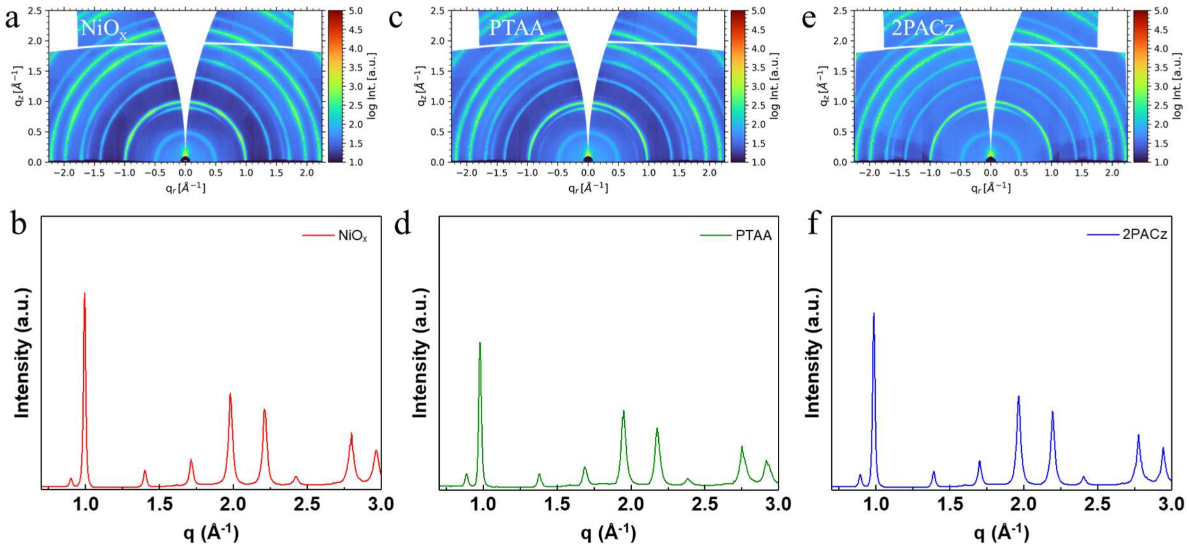

Figure S22 2D GIWAXS data of (a) NiO_x-, (c) PTAA-, and (e) 2PACz-based devices with an incidence angle of 0.2°. The correlation pseudo-XRD of (b) NiO_x-, (d) PTAA-, and (f) 2PACz-based devices.

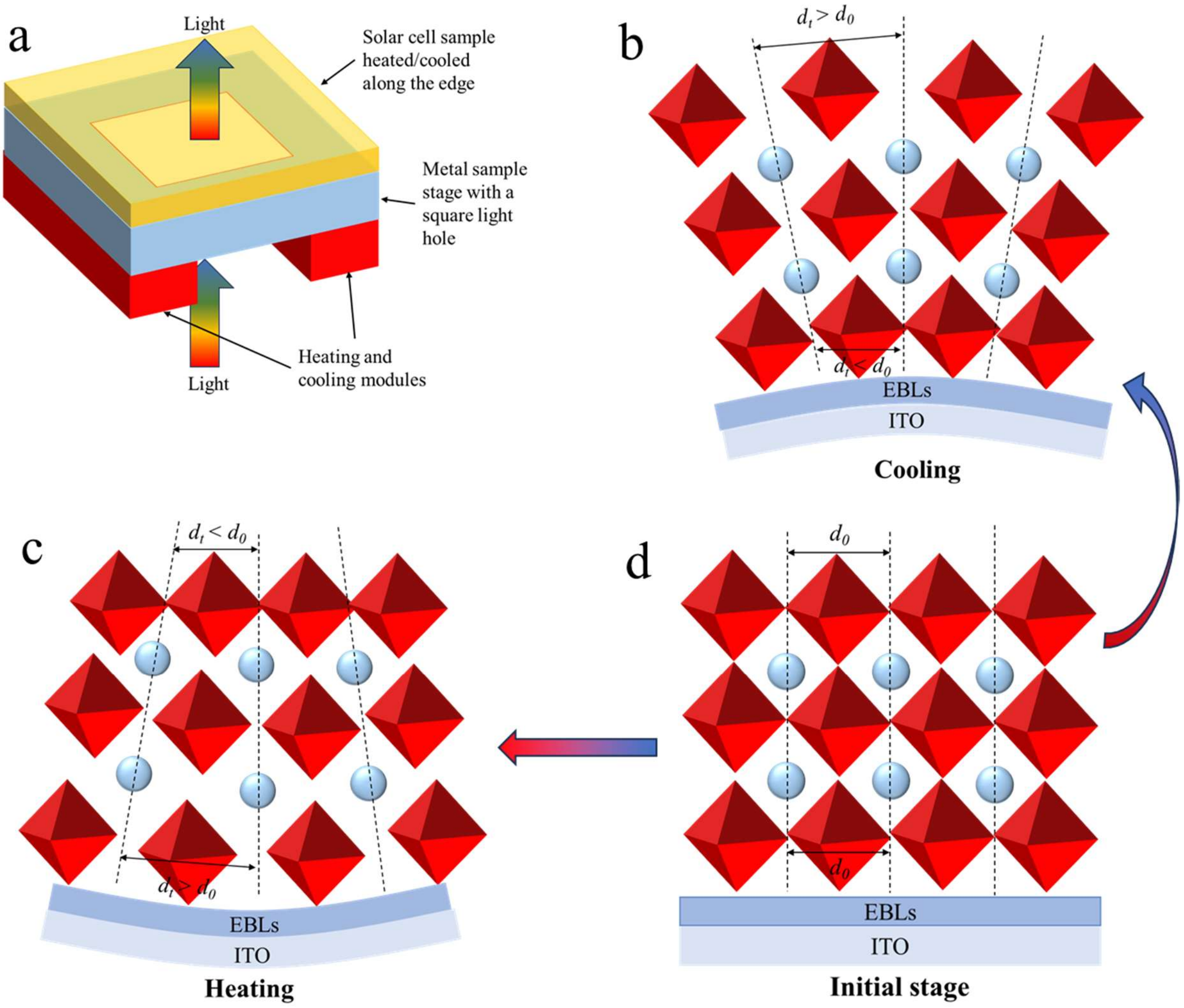

Figure S23 (a) Schematic diagram of the sample stage with a square light hole for the illuminating solar cells and a heating/cooling module for the temperature variation. Schematic illustration of the lattice expansion and shrinkage at the (b) cooling stage, (c) heating stage, and (d) initial stage.

References

[1] P. Zhu, D. Wang, Y. Zhang, Z. Liang, J. Li, J. Zeng, J. Zhang, Y. Xu, S. Wu, Z. Liu, X. Zhou, B. Hu, F. He, L. Zhang, X. Pan, X. Wang, N.-G. Park, B. Xu, Aqueous synthesis of perovskite precursors for highly efficient perovskite solar cells, *Science* **2024**, 383, 524.

[2] G. Benecke, W. Wagermaier, C. Li, M. Schwartzkopf, G. Flucke, R. Hoerth, I. Zizak, M. Burghammer, E. Metwalli, P. Müller-Buschbaum, A customizable software for fast reduction and analysis of large X-ray scattering data sets: applications of the new DPDAK package to small-angle X-ray scattering and grazing-incidence small-angle X-ray scattering, *Applied Crystallography* **2014**, 47, 1797.

[3] M. A. Reus, L. K. Reb, D. P. Kosbahn, S. V. Roth, P. Müller-Buschbaum, INSIGHT: in situ heuristic tool for the efficient reduction of grazing-incidence X-ray scattering data, *Applied Crystallography* **2024**, 57, 509.

[4] K. Sun, R. Guo, Y. Liang, J. E. Heger, S. Liu, S. Yin, M. A. Reus, L. V. Spanier, F. Deschler, S. Bernstorff, Morphological insights into the degradation of perovskite solar cells under light and humidity, *ACS Applied Materials & Interfaces* **2023**, 15, 30342.

[5] R. Guo, D. Han, W. Chen, L. Dai, K. Ji, Q. Xiong, S. Li, L. K. Reb, M. A. Scheel, S. Pratap, Degradation mechanisms of perovskite solar cells under vacuum and one atmosphere of nitrogen, *Nature energy* **2021**, 6, 977.

[6] H. Chen, T. Liu, P. Zhou, S. Li, J. Ren, H. He, J. Wang, N. Wang, S. Guo, Efficient bifacial passivation with crosslinked thioctic acid for high-performance methylammonium lead iodide perovskite solar cells, *Adv. Mater.* **2020**, 32, 1905661.

[7] J. Feng, C. H. Mak, G. Jia, B. Han, H. H. Shen, S. P. Santoso, J. J. Kai, M. Yuan, H. Song, J. C. Colmenares, H. Y. Hsu, Unlocking Interfacial Interactions of In Situ Grown Multidimensional Bismuth-Based Perovskite Heterostructures for Photocatalytic Hydrogen Evolution, *Adv. Energy Mater.* **2024**, 14, 2402785.

[8] X. Chen, K. Gao, X. Xu, L. Yang, S. Wang, W. Shi, F. Cao, W. Li, H. Li, Y. Li, B. Yang, C. Wang, W. Li, W. Wang, J. Xiao, S. Zhou, S. Yang, C. Yu, X. Zhang, X. Yang, Biased plasma treated nickel oxide for high-efficiency perovskite/silicon tandem solar cells,

Adv. Mater. **2025**, 37, 2504581.

[9] X. Jiang, S. Chen, Y. Li, L. Zhang, N. Shen, G. Zhang, J. Du, N. Fu, B. Xu, Direct surface passivation of perovskite film by 4-fluorophenethylammonium iodide toward stable and efficient perovskite solar cells, *ACS Appl. Mater. Interfaces* **2021**, 13, 2558.

[10] X. Chen, W. Feng, Y. Fang, Q. Li, Y.-H. Huang, X. Chang, C. Yao, Y. Shen, G. Liu, S. Yang, X.-D. Wang, M. Yuan, W.-Q. Wu, Improved conductivity of 2D perovskite capping layer for realizing high-performance 3D/2D heterostructured hole transport layer-free perovskite photovoltaics, *ACS Nano* **2025**, 19, 4299.
